# Supplementary material for: Self-assembled dendrimer polyamide nanofilms with enhanced effective pore area for ion separation
Source: Nat Commun. 2024 Jan 11;15:471. doi: 10.1038/s41467-023-44530-2 (PMC10784486; doi:10.1038/s41467-023-44530-2)
Supplement: Supplementary file 1 — Supplementary Information [file 41467_2023_44530_MOESM1_ESM.pdf]

Supplementary Information

Self-assembled dendrimer polyamide nanofilms with enhanced effective pore area for ion separation

Bingbing Yuan[1]*, Yuhang Zhang[1], Pengfei Qi[2], Dongxiao Yang[1], Ping Hu[1], Siheng Zhao[1,3], Kaili Zhang[1], Xiaozhuan Zhang[1], Meng You[1], Jiabao Cui[1], Juhui Jiang[1], Xiangdong Lou[1] and Q. Jason Niu[3]*

[1]School of Chemistry and Chemical Engineering, Key Laboratory of Green Chemical Media and Reactions Ministry of Education, Henan International Joint Laboratory of Aquatic Toxicology and Health Protection, Henan Normal University, 453007 Xinxiang, China.

[2]State Key Laboratory of Separation Membranes and Membrane Processes, National Center for International Research on Membrane Science and Technology, School of Materials Science and Engineering, Tiangong University, Tianjin 300387, P. R. China.

[3]Institute for Advanced Study, Shenzhen University, Nanshan District Shenzhen, 518060 Guangdong, China.

Correspondence and requests for materials should be addressed to Y. B. (email: yuanbingbing@htu.edu.cn) or to N. Q. J. (email: qjasonniu@szu.edu.cn)

List of Content:

1. Supplementary Methods
2. Supplementary Figures
3. Supplementary Tables
4. Supplementary Notes
5. Supplementary References

1. Supplementary Methods

1.1. Chemicals and materials

Trimesoyl chloride (TMC), piperazine (PIP), trifluoroacetic anhydride, 3,5-diaminobenzoic acid, sodium nitrite, P-phenylenediamine, hydrazine hydrate, diglycolic anhydride (DA), 1, 2, 4-benzeneticarboxylic anhydride (BA) and p-hydroxybenzoic acid were purchased from Tokyo Chemical Industry (Japan). G4 dendrimer (G4D) was synthetized based on our previous report[1,2]. Sodium hydroxide (NaOH), sodium chloride (NaCl), magnesium chloride hexahydrate ($MgCl_2$ $6H_2O$), anhydrous magnesium sulfate ($MgSO_4$), sodium sulfate (Na_2SO_4), calcium chloride ($CaCl_2$), sodium hydrogen carbonate ($NaHCO_3$), lithium chloride (LiCl), N-methyl-2-pyrrolidinone (NMP), thionyl chloride ($SOCl_2$), tetrachloroethane, dichloromethane, ethyl acetate, acetonitrile, cyclohexane, n-hexane, hydrochloric acid, polyethylene glycol (PEG), methanol, and N, N-dimethylformamide (DMF) were acquired from the Sinopharm Chemical Reagent Co, Ltd, and applied without further treatment. Polysulfone (PSF) support was provided by a commercial supplier (Blue Star Toray membrane. Corp.). DK and DL were obtained from Suez Environment. XC-N and NF 270 were acquired from DuPont. Deionized (DI) water (0.5–1.5 μs cm^{-1}) was prepared in a two-stage reverse osmosis purification system. Coverslips with a thickness ranging from 0.1 to 0.13 mm were purchased from Sail Brano Corp. and employed as a support for scanning electron microscopy (SEM).

1.2. Characterization methods

1.2.1. Nuclear magnetic resonance (NMR) spectroscopy

Approximately 15–20 mg of the prepared functional dendrimers (DA-G4D, BA-G4D and p-HC-G4D) and p-hydroxybenzoyl chloride (p-HC) was loaded into the NMR tube and dissolved with 2−3 mL $(CD_3)_2SO$ for ^1H NMR and ^{13}C NMR characterization. All data were analyzed with MestReNova software.

1.2.2. High-resolution mass spectrometry (HRMS)

4-6 mg dendrimers were dissolved in DMF and 0.0076 mM was used as an additive, then the resulting liquid was diluted to a certain multiple with DMF. After that, taking

acetonitrile and water as mobile phase, Electrostatic Field Orbitrap Ultra High-Resolution Liquid Chromatography Mass Spectrometer (Orbitrap Exploris MX, Thermo Fisher Scientific, made in Germany) was applied to identity molecular weight of the synthetic dendrimers.

1.2.3. Brunauer Emmett Teller (BET)

The specific surface area of DA-G4D, BA-G4D and p-HC-G4D were identified by the pore volume as determined by the Brunauer Emmett Teller (BET) method (ASAP2020 specific surface area and pore analyzer).

1.2.4. Scanning electron microscopy observation (SEM)

Thin nanofilm composite membranes are consisted of three layers, an ultrathin polyamide top layer, a PSF support, and a nonwoven fabric. For accurately observing the cross-sectional morphology of the polyamide nanofilm, the nonwoven fabric was first peeled off by using adhesive tape. Then, the remaining PSF support with polyamide layer was soaked in DMF until the polyamide became fully transparent, indicating that the PSF support material was no longer present, and then washed with methanol. For the cross-sectional morphology, the polyamide layers without PSF support were deposited onto the coverslips by a floating method and fractured in liquid nitrogen for scanning electron microscopy observation (SEM, Hitachi SU8010). The samples were coated with gold before SEM analysis.

1.2.5. Transmission electron microscope (TEM) and High-resolution transmission electron microscope (HRTEM)

Morphology of the functional dendrimers (DA-G4D, BA-G4D and p-HC-G4D) and the self-assembled dendrimers (SADs) were observed by HRTEM (TEM, JEM-1200EX, JEOL). The dissolved functional dendrimers and SADs solutions were deposited onto the copper meshes for HRTEM characterizations. The SADs polyamide nanofilms without PSF support were deposited onto the copper meshes for the inner morphology observation with TEM (TEM, JEM-1200EX, JEOL).

For the cross-sectional morphology of the SADs polyamide nanofilms, the samples were firstly embedded with resin and then made ultrathin section. Specifically, the

membrane samples were performed gradual dehydration with a series of ethanol aqueous solution, such as 0%, 25%, 50%, 75% and 100% ethanol content, 5 minutes per step. Then, those samples were embedded into resin (LR White Resin, London Resin Company, Reading, UK). Resin embedded membrane samples were cut into ultra-thin slices of approximately 80 nm using Leica EM UC7 (Leica Microsystems Wetzlar, Germany). The slices were placed onto the copper meshes for TEM characterizations (JEM1200EX TEM, accelerating voltage 80 kV JEOL, Japan).

1.2.6 X-ray photoelectron spectroscopy (XPS)

The chemical composition and structure of the SADs polyamide nanofilms were characterized by XPS. The chemical composition and elemental data obtained from XPS were analyzed and fitted using CasaXPS software.

1.2.7 Zeta potential

Surface charge of the SADs polyamide nanofilms was determined with an Anton Paar SurPass solid surface analyzer. Test condition: 1 mM KCl, 25°C.

1.2.8 Water contact angle

Water contact angle was measured under room temperature (25°C) using a drop shape Analyzer-DSA30 (KRÜSS, Germany) in the sessile drop mode for characterizing the surface hydrophilicity of the membrane surface.

1.3 Supplementary Experimental

1.3.1 Synthesis steps of G4 dendrimer (G4D)

Synthesis steps of 3, 5-bis (N-trifluoro acetamido) benzoic acid

Trifluoroacetic anhydride (16.51 g, 78.6 mmol) was added to a 30 mL THF solution containing 3,5-diaminobenzoic acid (3.65 g, 24 mmol) at 0°C under nitrogen, and stirred at that temperature for 15 min. Subsequently, the system was stirred for 3 h at 25°C oil bath. Then, water (30 mL) was added and continued to be stirred for 6 h. The resulted mixture was extracted with the ethyl acetate to obtain the organic layer and aqueous layer. The organic layer was washed with water 3–4 times, and dried with anhydrous magnesium sulfate overnight. Afterwards, the resulted filtrate was evaporated to give a purple powdery solid, and recrystallized from acetonitrile, filtrated

and gave purple solid particles. The product was dried at 120°C for 12 h to give pale purple solid particles (6.1 g, a yield of 79%).

Synthesis steps of 3, 5-bis (trifluoro acetamido) benzoyl chloride

A 150 mL of thionyl chloride solution containing 3, 5-bis (N-trifluoro acetamido) benzoic acid (16.03 g, 46.6 mmol) was refluxed for 6 h at 120°C. The thionyl chloride is distilled off and the residue was dissolved in 1, 1, 2, 2-tetrachloroethane at 100°C, Subsequently, cooled to room temperature to precipitate a purple solid powder. The solid was washed with n-hexane three times to give a brown powder. Afterwards, the power was recrystallized with dichloromethane, dried at 60°C to give slightly yellow power (12.1 g, a yield of 72%).

Synthesis steps of G1 dendrimer (G1D)

3,5-bis (N-trifluoro acetamido) benzoic chloride (7.963 g, 22 mmol) was added to a 10 mL of NMP solution containing p-Phenylenediamine (1.08 g, 10 mmol), stirred for 15 min at 0°C, and subsequently stirred for 1 h at 25°C oil bath. The water (50 μL) was added to the system and reacted for 1.5 h at 50°C. Then, hydrazine hydrate (6 g, 120 mmol) was added dropwise and continued to be stirred for 1.5 h. The reaction solution was poured into a 100 mL of 2 wt% $NaHCO_3$ solution, stirred for 30 min, filtered under suction, and dried to give light gray solid of G1D (7.4 g, yield 98%).

Synthesis steps of G2 dendrimer (G2D)

3,5-bis (N-trifluoro acetamido) benzoic chloride (7.963 g, 22 mmol) was added to a 20 mL of NMP solution containing G1D (1.88 g, 5.0 mmol), stirred for 15 min at 0°C, and subsequently stirred for 1 h at 25°C oil bath. The water (50 μL) was added to the system and reacted for 1.5 h at 50°C. Then, hydrazine hydrate (6 g, 120 mmol) was added dropwise and continued to be stirred for 1.5 h. The reaction solution was poured into a 100 mL of 2 wt% $NaHCO_3$ solution, stirred for 30 min, filtered under suction, and dried to give light gray solid of G2D (4.4 g, yield 97%).

Synthesis steps of G3 dendrimer (G3D)

3,5-bis (N-trifluoro acetamido) benzoic chloride (7.963 g, 22 mmol) was added to a 27 mL of NMP solution containing G2D (2.28 g, 2.5 mmol), stirred for 15 min at 0°C,

and subsequently stirred for 1 h at 25°C oil bath. The water (50 μL) was added to the system and reacted for 1.5 h at 50°C. Then, hydrazine hydrate (6 g, 120 mmol) was added dropwise and continued to be stirred for 3.5 h. The reaction solution was poured into a 133 mL of 2 wt% $NaHCO_3$ solution, stirred for 30 min, filtered and dried to give light gray solid of G3D (4.7 g, yield 94%).

Synthesis steps of G4 dendrimer (G4D)

3,5-bis (N-trifluoro acetamido) benzoic chloride (7.963 g, 22 mmol) was added to a 27 mL of NMP solution containing G3D (2.581 g, 1.3 mmol), stirred for 15 min at 0°C, and subsequently stirred for 1 h at 25°C oil bath. The water (50 μL) was added to the system and reacted for 1.5 h at 50°C. Then, hydrazine hydrate (6 g, 120 mmol) was added dropwise and continued to be stirred for 3.5 h. The reaction solution was poured into a 133 mL of 2 wt% $NaHCO_3$ solution, stirred for 30 min, filtered and dried to give light gray solid of G4D (4.8 g, yield 92%).

1.3.2 Chemical structure of DA-G4D dendrimer

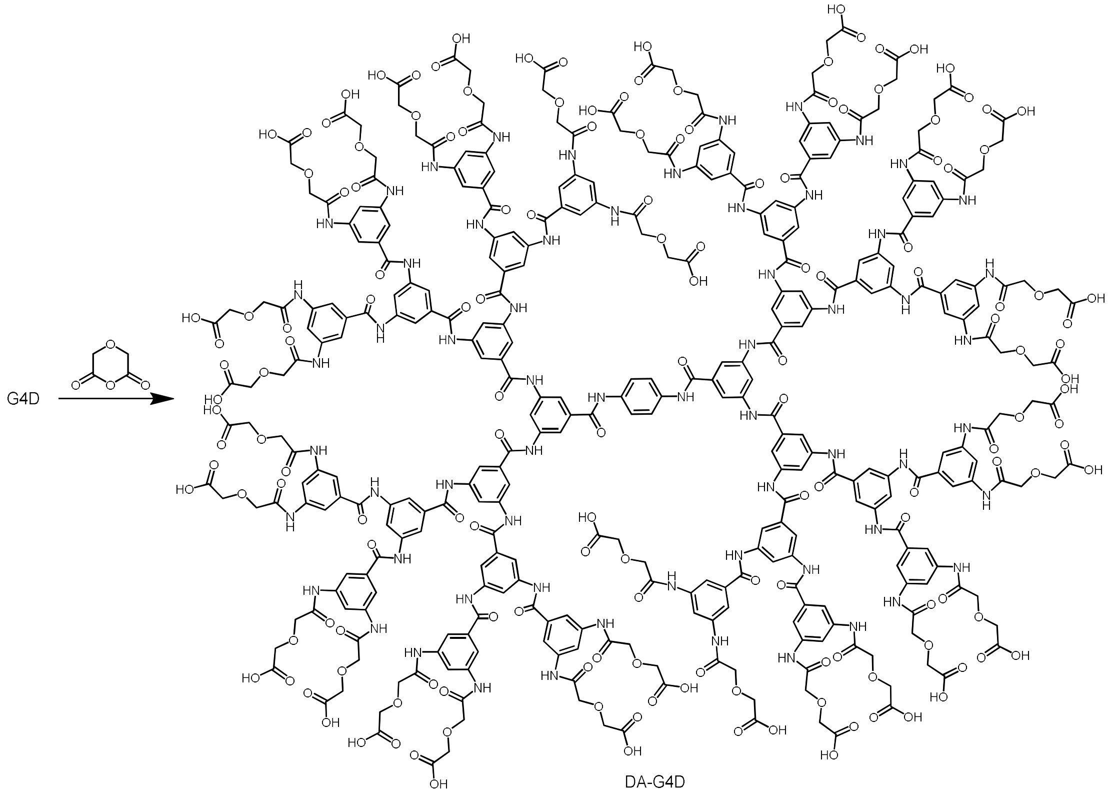

DA-G4D

Supplementary Figure 1 | Chemical structure of DA-G4D dendrimer.

1.3.3 Chemical structure of BA-G4D dendrimer

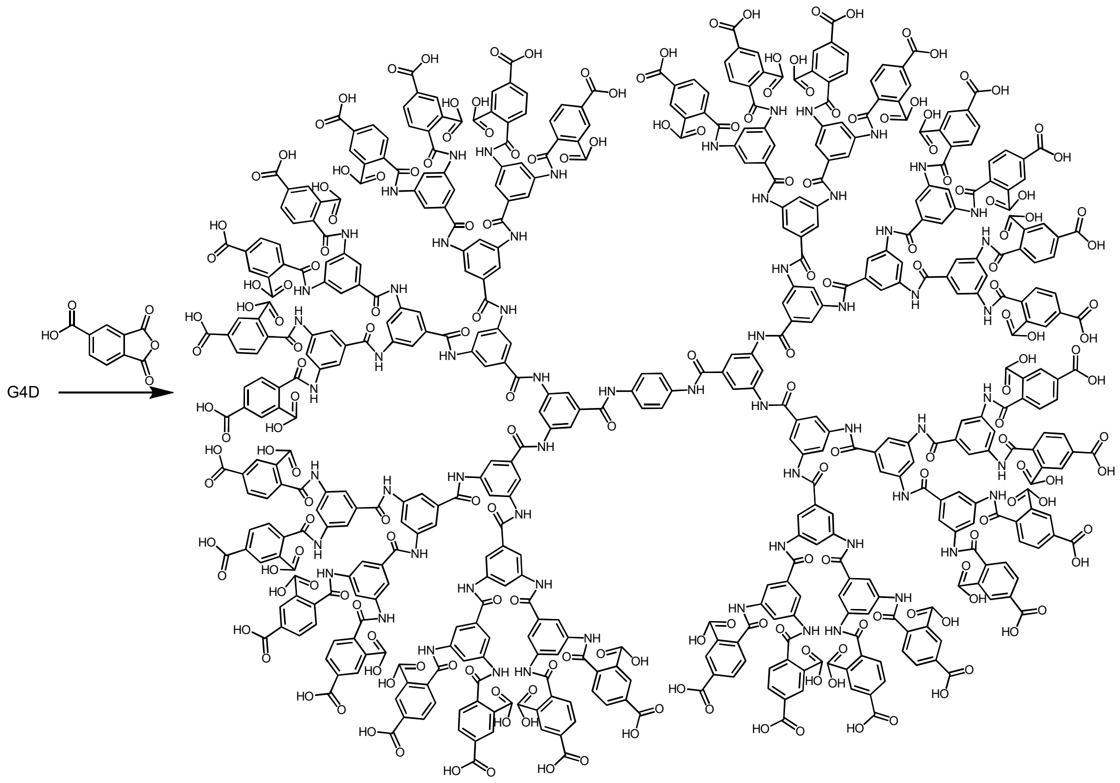

BA-G4D

Supplementary Figure 2 | Chemical structure of BA-G4D dendrimer.

1.3.4 Chemical structure of p-hydroxybenzoyl chloride (p-HC) and p-HC-G4D dendrimer

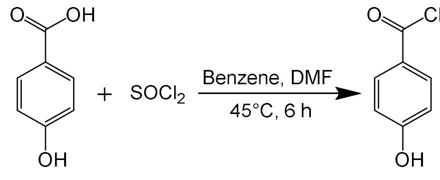

p-HA p-HC

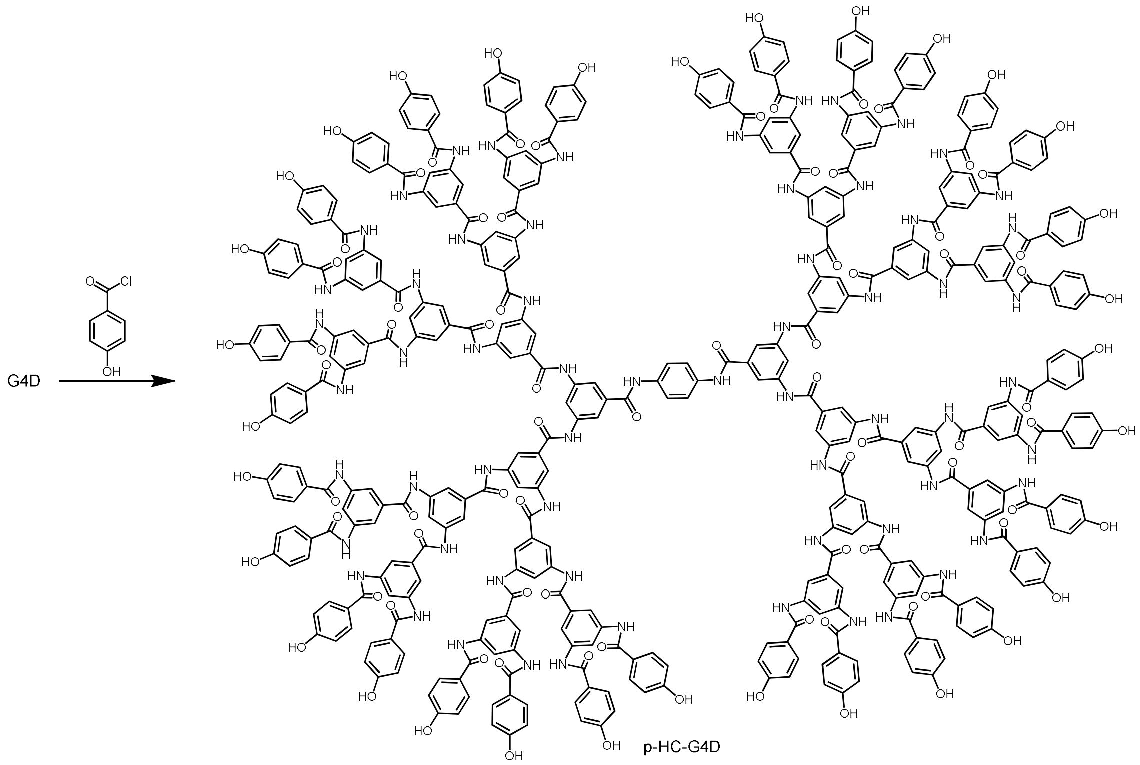

Supplementary Figure 3 | Chemical structure of p-HC-G4D dendrimer.

1.3.5 Membrane performance test

Desalination performance of the prepared polyamide membrane was determined with different salt solutions in a cross-flow system with an effective test area (A) of 19.3 cm^2. The concentration of the single salt, such as $MgCl_2$, LiCl, $CaCl_2$, $MgSO_4$, Na_2SO_4, and NaCl, in the feed solution was 2 g L^{-1} (2000 ppm). Test condition: 1 MPa or 1.5 MPa, a temperature of 25°C. The performance data were determined after the water flux and the conductivity reached a steady state. For the stability test, the SADs polyamide membranes were continuously operated for 24 h at 1 MPa, 25°C. The water flux (L m^{-2} h^{-1}) was calculated from the volume of the permeate (M) for a specified time, as given by the following equation:

$$\text{Water Flux (kg m}^{-2}\text{ h}^{-1}) = M/At \qquad (1)$$

The salt rejection was determined from the conductivity of the feed solution (C_f) and the permeate (C_p). Hence, the salt/ion rejection can be calculated from the following equation:

$$\text{Rejection (\%)} = \left(1\text{-}C_p/C_f\right)\times 100\% \qquad (2)$$

The ion selectivity of the Li^+/Mg^{2+}, Cl^-/SO_4^{2-} for the resulted membranes were conducted in the mixed salt solution such as $LiCl/MgCl_2$, $NaCl/Na_2SO_4$. The ion concentrations of the permeation and the feed were measured via ion chromatograph (Thermo Dionex Aquion). The ion rejection was calculated through Equation 1. Based on Equation 3, the corresponding separation factor was obtained.

$$\alpha=\frac{C_{A,\ p}}{C_{A,f}}\frac{C_{B,\ f}}{C_{B,p}}=\frac{100-R_A}{100-R_B} \quad (3)$$

where $C_{A,p}$ is the concentration of the Cl^- or Li^+ in the permeate, $C_{A,f}$ is the concentration of the Cl^- or Li^+ in the feed, $C_{B,p}$ is the concentration of the SO_4^{2-} or Mg^{2+} in the permeate and $C_{B,f}$ is the concentration of the SO_4^{2-} or Mg^{2+} in the feed. R_A is the rejection rate of the Cl^- or Li^+, and R_B is the rejection rate of the SO_4^{2-} or Mg^{2+}.

1.3.6 Mean effective pore size and pore size distribution

PEG molecules were used as the neutral solute to obtain the rejection curves and the molecular weight cut-off (MWCO). Molecular weight of the PEG molecules used were 200 Da, 400 Da, 600 Da and 800 Da. Test conditions are as follows: 1 MPa, 0.2 g L^{-1} PEG molecules, 7.5 L min^{-1} and 25 °C. The pore size distribution and mean effective pore size was calculated according to the previous reported method[3]. The Stokes radius of different neutral molecules used was calculated based on Equation 4.

$$r_s\ (nm)=1.674\times10^{-4}\times Mw^{0.557} \quad (4)$$

As illustrated in Equation 5, the neutral solution rejection can function as the solution radius.

$$R_T=\mathrm{erf}(y)=\frac{1}{\sqrt{2\pi}}\int_{-\infty}^{y} e^{-(u^2/2)}\, d_u,\ \text{where}\ y=\frac{\ln r_s-\ln \mu_s}{\ln \sigma_g} \quad (5)$$

Of which, R_T and r_s are the rejection rate and radius of the neutral molecules, μ_s is the Stokes radius of PEG molecules at R_T = 50%, while σ_g is the ratio of radius of the neutral molecule at R_T = 84.13% and 50%. When the solution rejection has functioned as the radius, a straight line related to log-normal probability coordinates can be obtained, as shown in Equation 6.

$$F(R_T)=A+B(\ln r_s) \quad (6)$$

Finally, by ignoring the effects of hydrodynamic interactions between the nanopore of PA nanofilm and the neutral solution radius, the geometric standard deviation (σ_p) and the mean effective pore radius (μ_p) can be supposed as the same with the μ_s and σ_g. Hence, combined Equations 4, 5 and 6, the pore size distribution enables to be illustrated as Equation 7.

$$\frac{dR_T(r_p)}{dr_p}=\frac{1}{r_p \ln\sigma_p\sqrt{2\pi}}\exp-\frac{\left(\ln r_p-\ln\mu_p\right)^2}{2\left(\ln\sigma_p\right)^2} \quad (7)$$

Of which, r_p is the pore radius that can be effectively intercept the salt, ion, or neutral molecules.

1.3.7 Density measurement

Densities of SADs polyamide nanofilms were measured and calculated by the ellipsometry (J. A. Woollam Co., Lincoln, NE) and thickness from the SEM characterizations. The floating method was used to isolate and deposit the resulted nanofilm onto the QCM sensors, and then analyzed the change in the frequency of vibration of QCM sensors to obtain the surface density of the samples. By dividing the areal density by the nanofilm thickness (from the SEM results), we can calculate the layer density. For accuracy, five locations on each sample were analyzed.

1.3.8 Calculation of volumetric charge density

According to the method reported by Shardul S. Wadekar and Radisav D. Vidic[4], using Equations (8) and (9), we calculated the σ_{ek}, electrokinetic charge density (C/m^2), and $-X$, volumetric charge densities (mol m^{-3}):

$$\sigma_{ek}=-\operatorname{sign}(\zeta)\sqrt{\left(2\varepsilon_0\varepsilon_b RT\sum_i c_i^b\left[\exp\left(\frac{-z_iF\zeta}{RT}\right)-1\right]\right)} \quad (8)$$

$$-X=\frac{\sigma_{ek}}{\gamma_p F} \quad (9)$$

Of which, the γ_p is the same as the μ_p (mean effective pore radius), ε_0 is vacuum permittivity (8.854×10^{-12} C/(mV)), ε_b is dielectric constant for the feed salt solution (assumed to be equal to that of water at 25 °C=80.1), R is universal gas constant

(8.31446 J/(mol·K)), T is absolute temperature (296.15 K), c_i^b is bulk feed concentration of ion i, z_i is valence of ion i, F is Faraday constant (96485.3329 C/mol).

1.3.9 Adsorption behaviors of NaCl

For further inspection on the nanopore structure of the SADs polyamide nanofilms, we used Quartz Crystal Microbalance (QCM) to study their adsorption behaviors on NaCl. To be specific, commercial gold-coated quartz crystal sensors with the frequency of 4.95 MHz were first cleaned by UV/Ozone for 10 min, followed by deionized water rinsing and being dried by ultrapure N_2. The resulted nanofilms were deposited onto the gold-coated sensor and dried at 50°C overnight. To measure the deposition kinetics of NaCl, the polyamide-coated sensor was placed in the QCM-D chamber. The sensor was first established by air. Deionized water was then pumped into the chamber at a flow rate of 50 μL min^{-1}. After stabilizing the frequency and dissipating for 2 hours with deionized water, a 50 ppm NaCl solution was introduced into the polyamide surface at a constant flow rate of 50 μL min^{-1}. After 2 hours of adsorption, 100 ppm NaCl solution was introduced into the polyamide surface, and then after 2 hours of adsorption and penetration, 200 ppm NaCl solution was introduced into the polyamide surface. QCM-D determined the changes of the oscillation frequency (f) and energy dissipation (D) of the quartz crystal sensor due to the adsorption of NaCl in the polyamide nanofilms.

2 Supplementary Figures

2.1 Characterization of DA-G4D, BA-G4D and p-HC-G4D

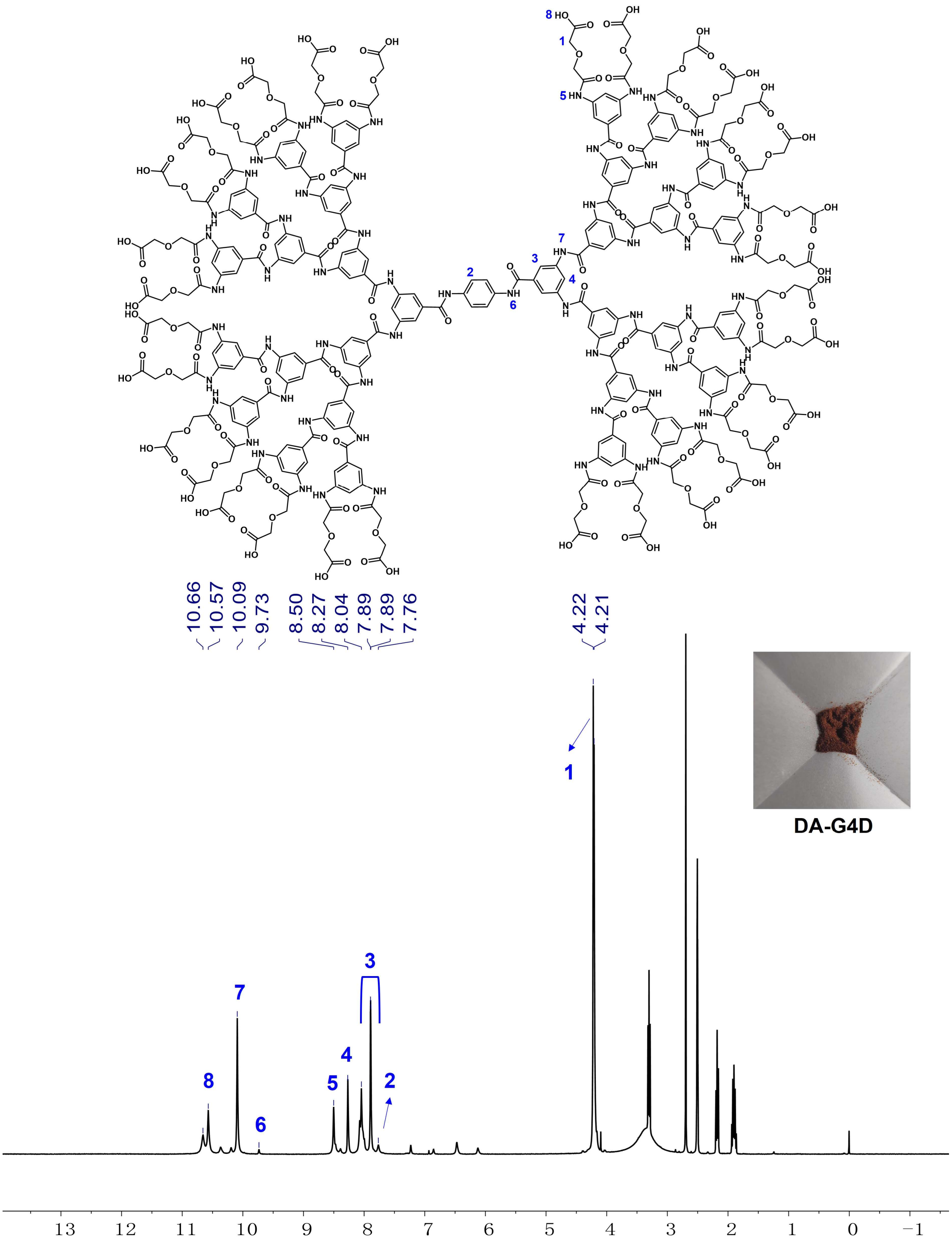

Supplementary Figure 4 | ^{1}H NMR spectrum of DA-G4D in $(CD_3)_2SO$. ^{1}H NMR (400 MHz, $(CD_3)_2SO$): δ 4.21-4.22 (128H), 7.76 (4H), 7.89–8.04 (60H), 8.27 (30H), 8.50 (32H), 9.73 (2H), 10.09 (28H), 10.57–10.36 (32H).

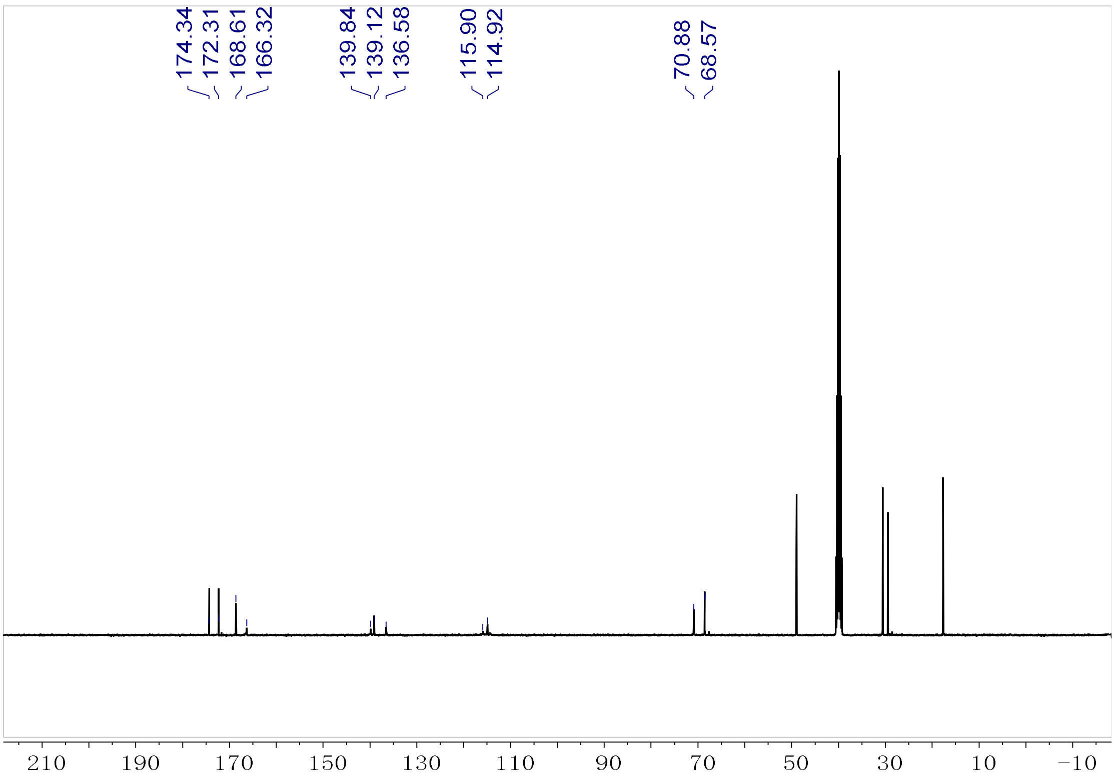

Supplementary Figure 5 | ^{13}C NMR spectrum of DA-G4D in $(CD_3)_2SO$. ^{13}C NMR (400 MHz, $(CD_3)_2SO$): δ 68.57, 70.88, 114.92, 115.9, 136.58, 139.12, 139.84, 166.32, 168.61, 172.31, 174.34.

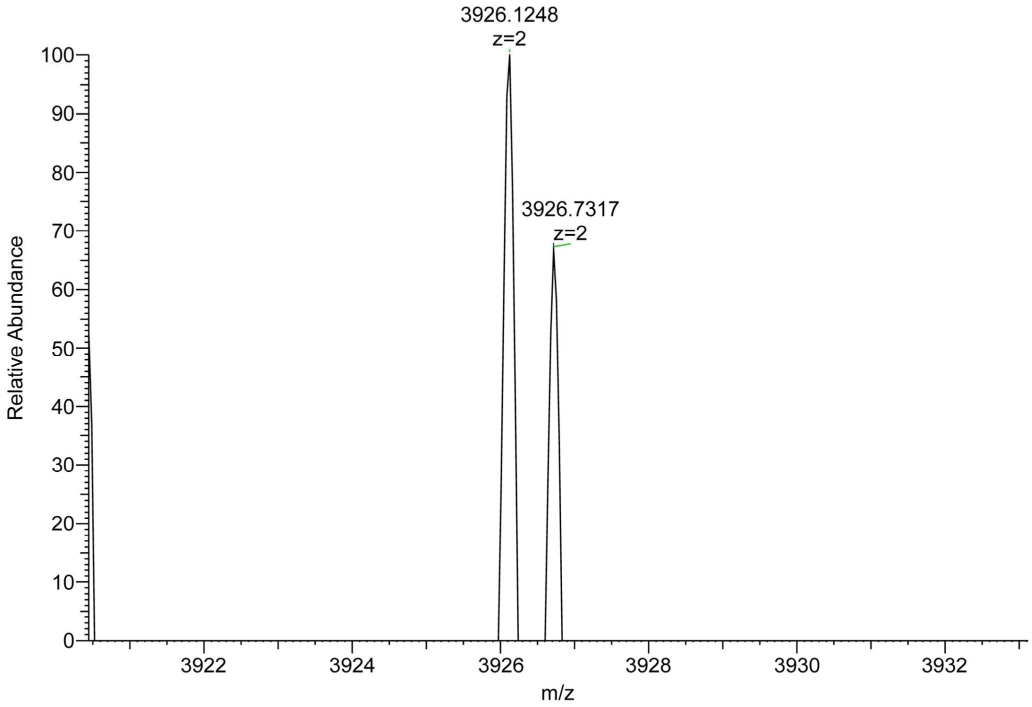

Supplementary Figure 6 | High-resolution mass spectral (HRMS) of DA-G4D. $[M]^+$=7846.59; 1, 0.5$[M+6H]^+$=3926.12; 2, 0.5$[M+6H]^+$=3926.73.

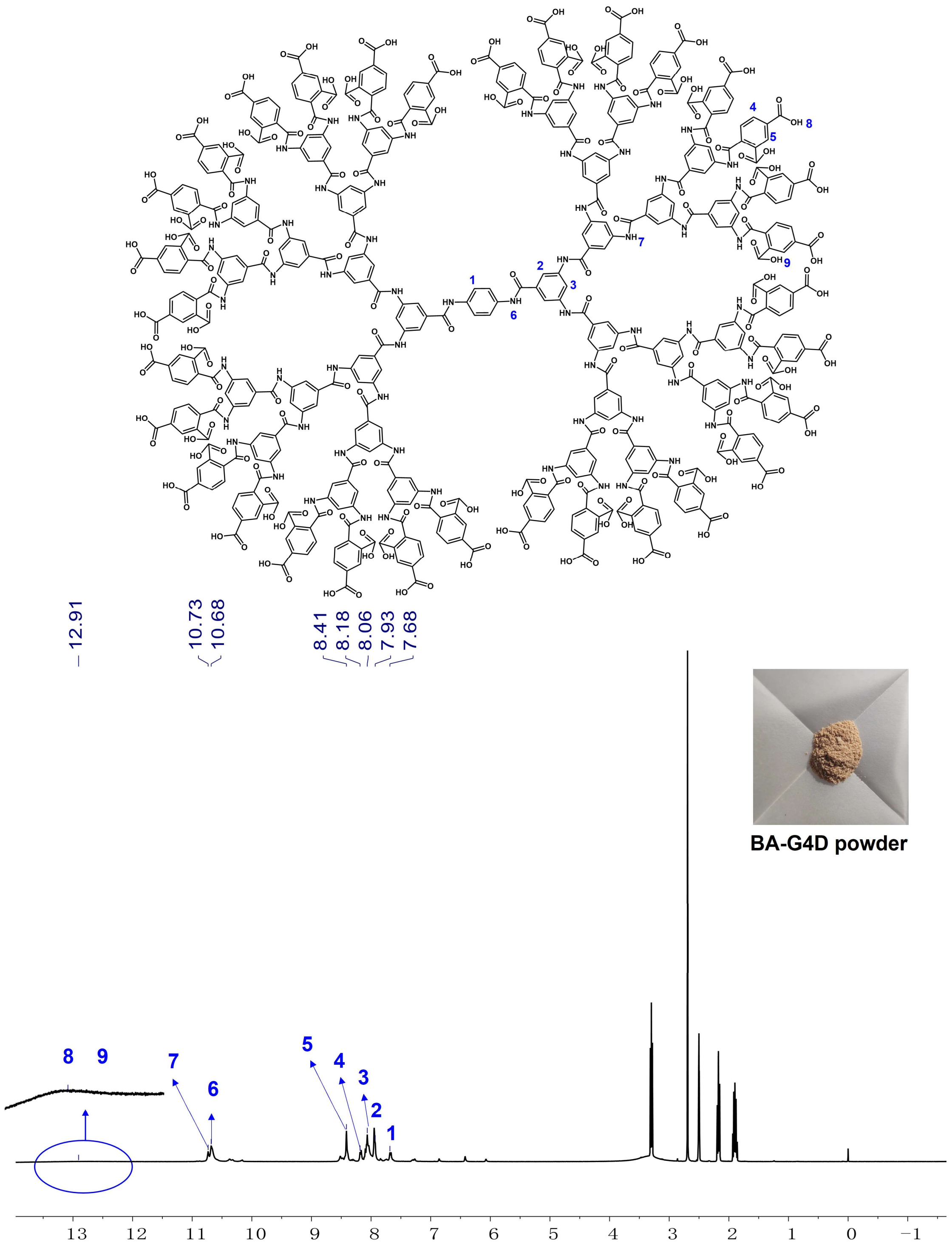

Supplementary Figure 7 | ^{1}H NMR spectrum of BA-G4D in $(CD_3)_2SO$. δ 7.68 (4H), 7.93 (60H), 8.06 (30H), 8.18 (32H), 8.41 (32H), 10.68 (2H), 10.73 (60H), 12.91 (64H).

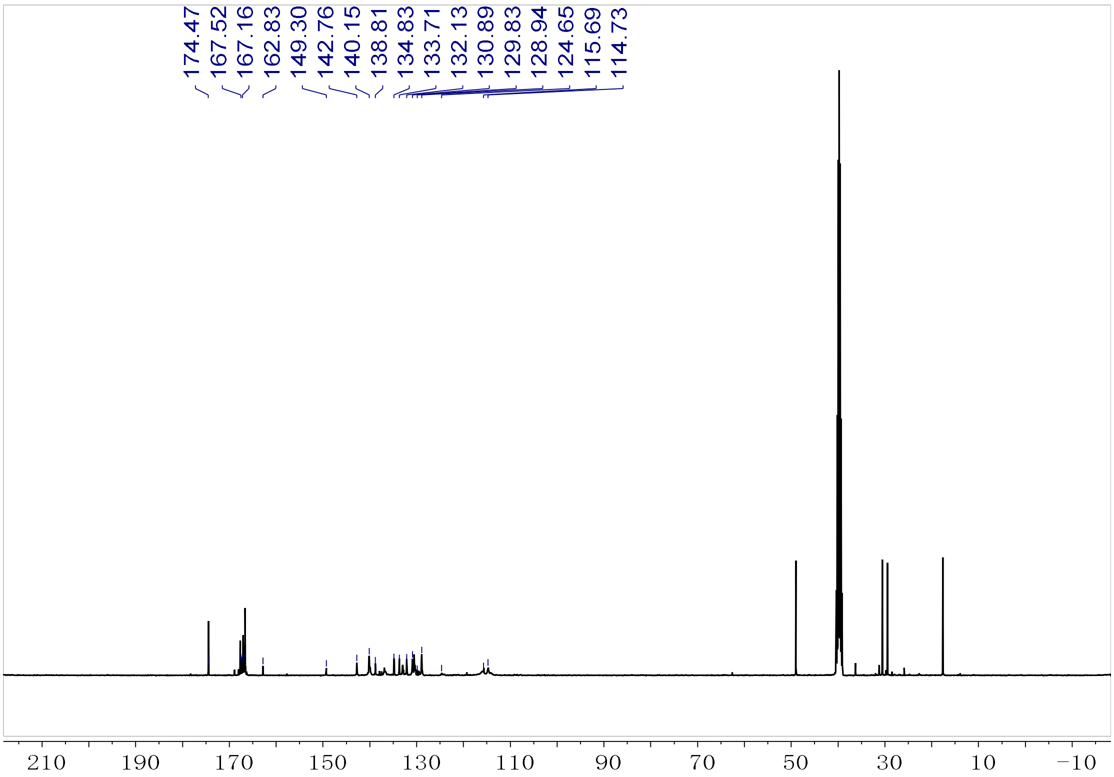

Supplementary Figure 8 | ^{13}C NMR spectrum of BA-G4D in $(CD_3)_2SO$. ^{13}C NMR (400 MHz, $(CD_3)_2SO$): δ 114.73, 115.69, 124.65, 128.94, 129.83, 130.89, 132.13, 133.71, 134.83, 138.81, 140.15, 142.76, 149.30, 162.83, 167.16, 167.52, 174.47.

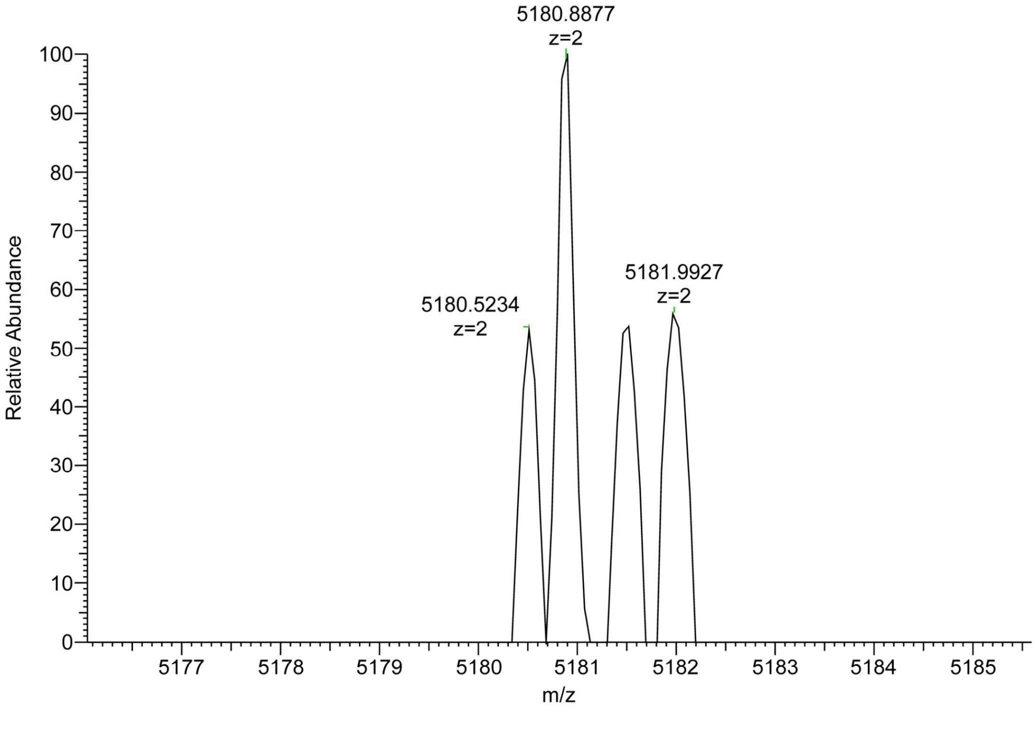

Supplementary Figure 9 | HRMS of BA-G4D. $[M]^+$=10280.32; 1, 0.5$[M+2K+2H]^+$=5180.52; 2, 0.5$[M+2K+2H]^+$=5180.89; 3, 0.5$[M+2K+4H]^+$=5181.99.

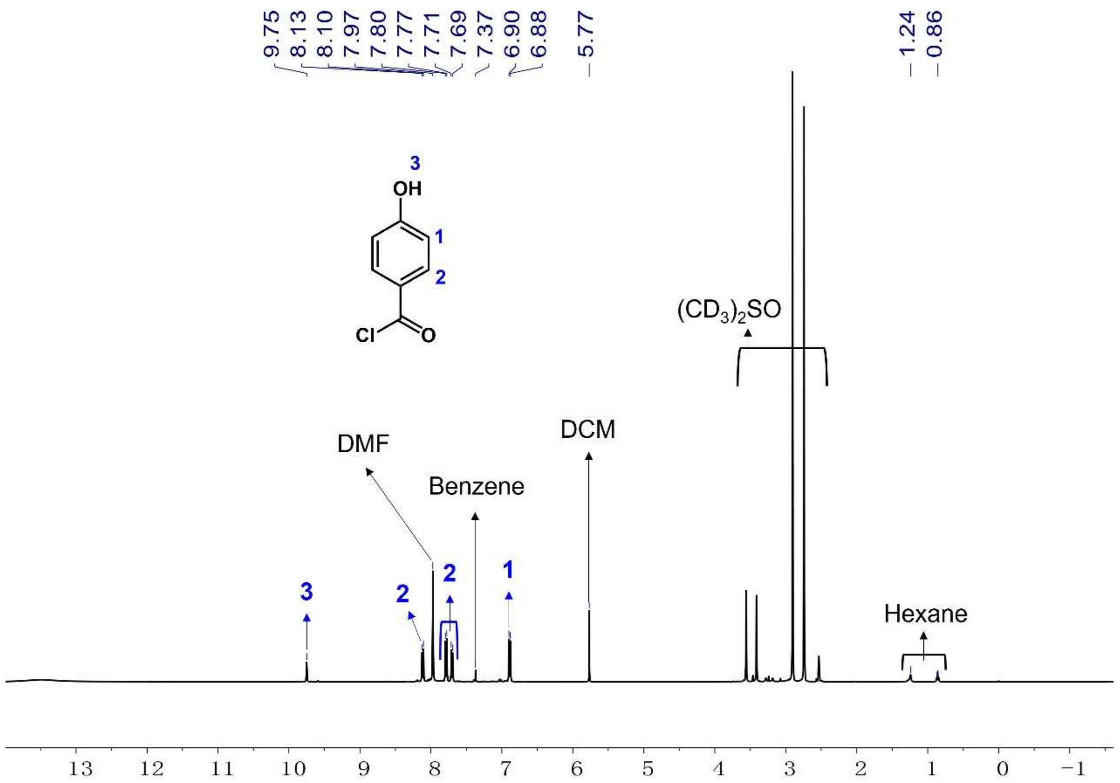

Supplementary Figure 10 | ^{1}H NMR spectrum of p-Hydroxybenzoyl chloride in $(CD_3)_2SO$.

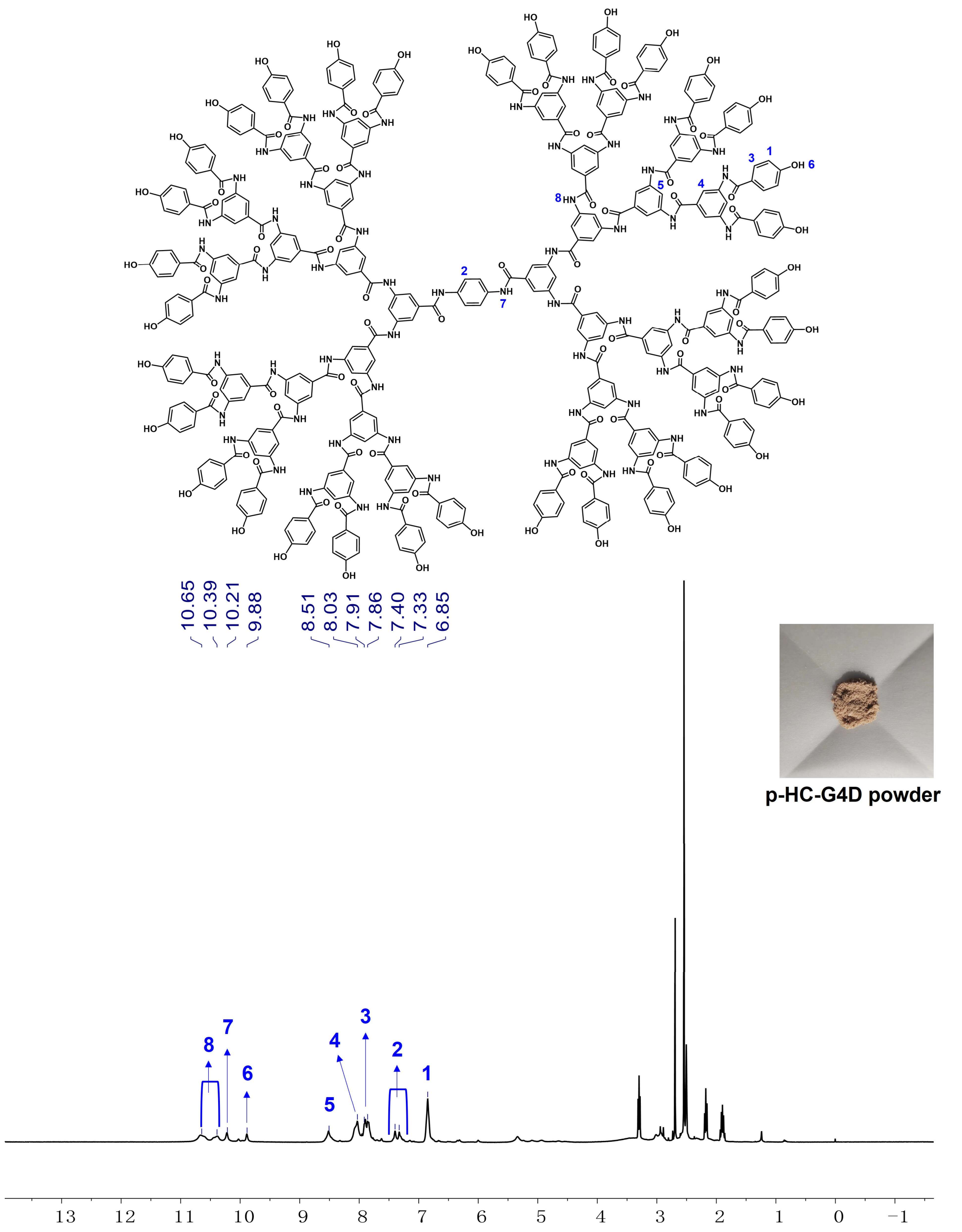

Supplementary Figure 11 | ^{1}H NMR spectrum of p-HC-G4D in $(CD_3)_2SO$. ^{1}H NMR (400 MHz, $(CD_3)_2SO$): δ 6.85 (64H), 7.33–7.40 (4H), 7.86–7.91 (64H), 8.03 (32H), 8.51 (8H), 9.88 (32H), 10.39 (2H), 10.39–10.65 (60H).

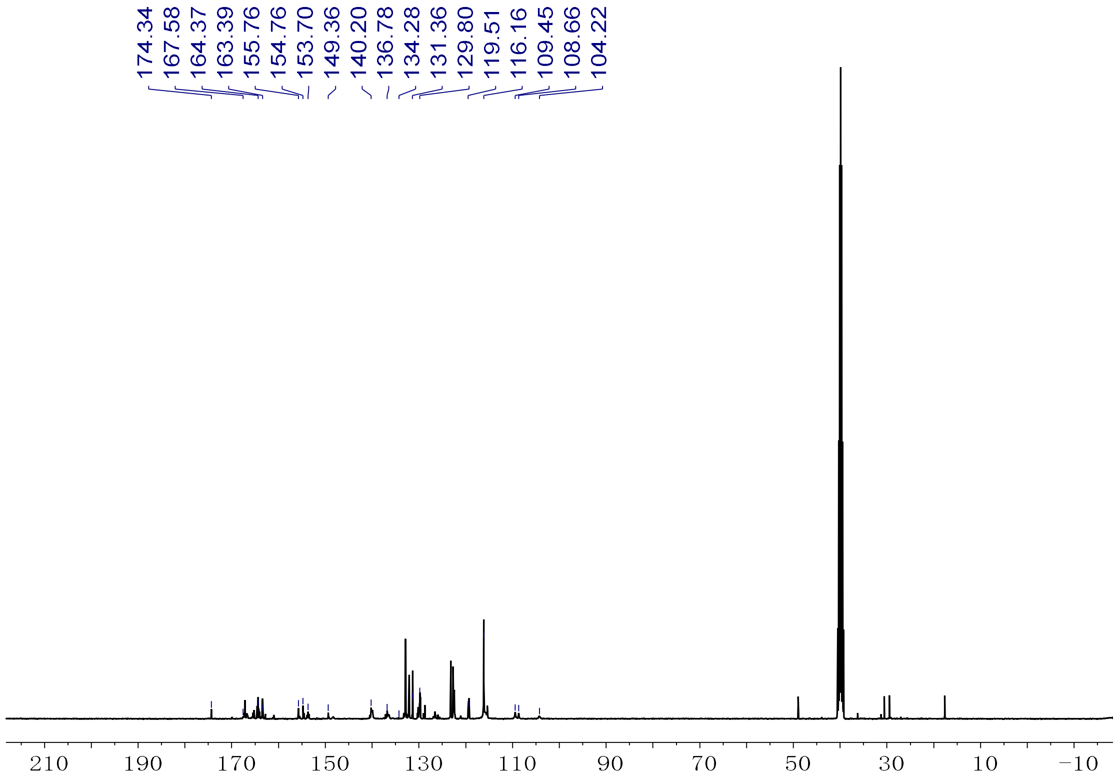

Supplementary Figure 12 | ^{13}C NMR spectrum of p-HC-G4D in $(CD_3)_2SO$. ^{13}C NMR (400 MHz, $(CD_3)_2SO$): δ 104.22, 108.66, 109.45, 116.16, 119.51, 129.80, 131.36, 134.28, 136.78, 140.20, 149.36, 153.70, 154.76, 155.76, 163.39, 164.37, 167.58, 174.34.

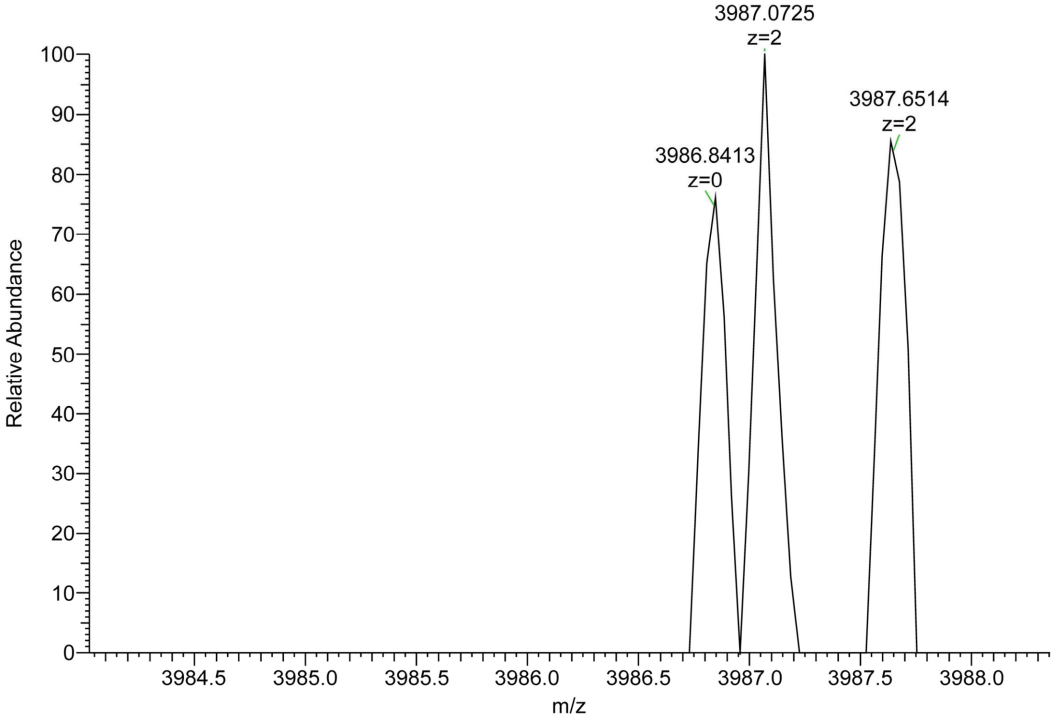

Supplementary Figure 13 | HRMS of p-HC-G4D. $[M]^+$= 7975.71; 1, 0.5$[M-2H]^+$=3986.84; 2, 0.5$[M]^+$=3987.65.

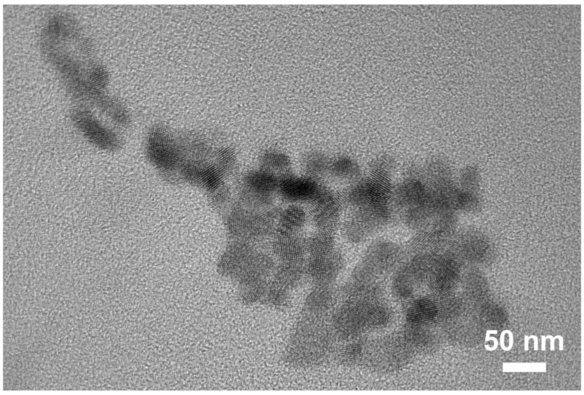

Supplementary Figure 14 | Morphology of the DA-G4D from HRTEM images.

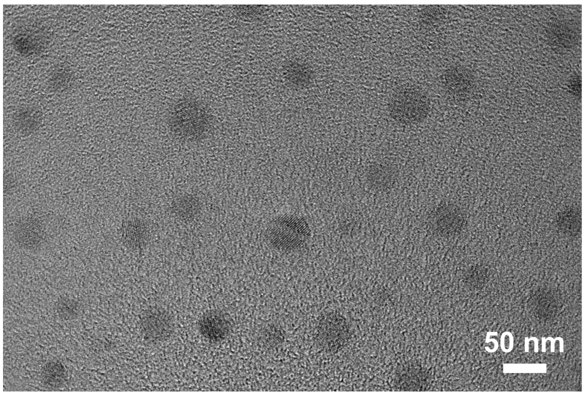

Supplementary Figure 15 | Morphology of the BA-G4D from HRTEM images.

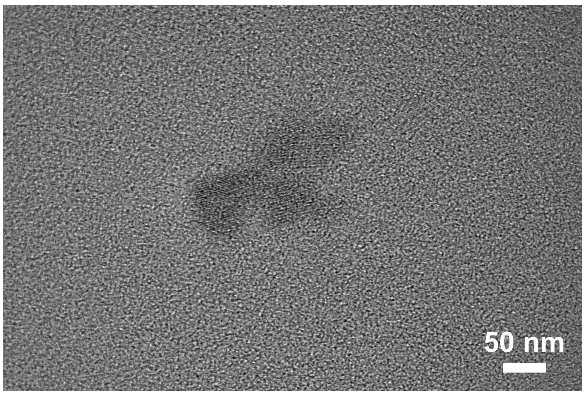

Supplementary Figure 16 | Morphology of the p-HC-G4D from HRTEM images.

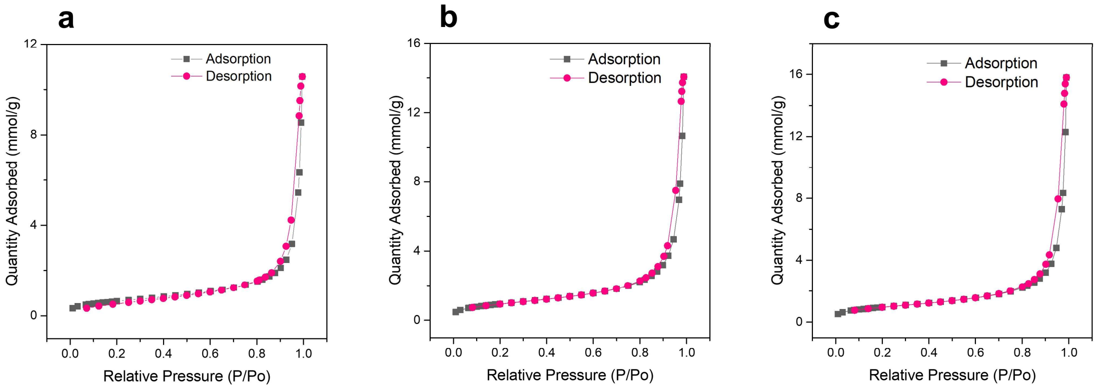

Supplementary Figure 17 | N_2 sorption at 77 K for the DA-G4D (a), BA-G4D (b) and p-HC-G4D (c).

As shown in Supplementary Figure 17, according to BET (Brunauer Emmet Teller) N_2 adsorption–desorption experiments, the surface area of DA-G4D, BA-G4D and p-HC-G4D is as high as 50.1465 $m^2\ g^{-1}$, 75.7204 $m^2\ g^{-1}$, and 76.5585 $m^2\ g^{-1}$.

2.2 Formation of self-assembled dendrimers

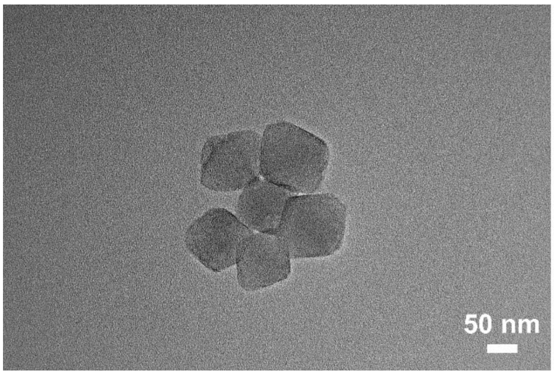

Supplementary Figure 18 | Morphology of the DA-G4D SADs from HRTEM images.

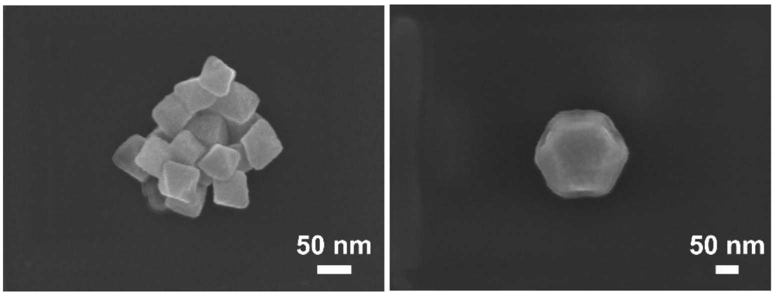

Supplementary Figure 19 | Morphology of the DA-G4D SADs from SEM images.

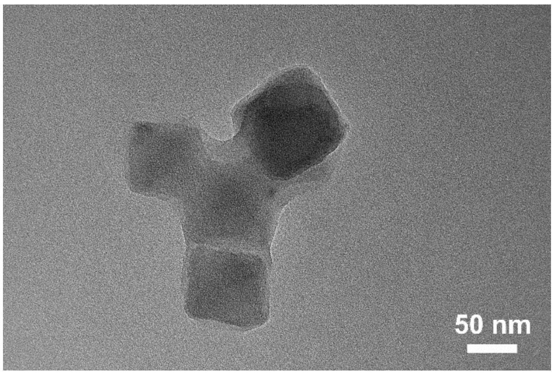

Supplementary Figure 20 | Morphology of the BA-G4D SADs from HRTEM images.

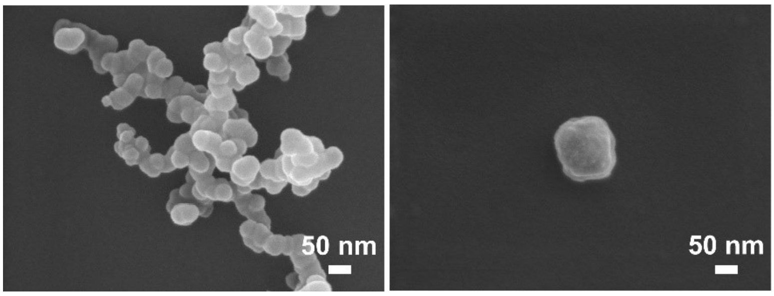

Supplementary Figure 21 | Morphology of the BA-G4D SADs from SEM images.

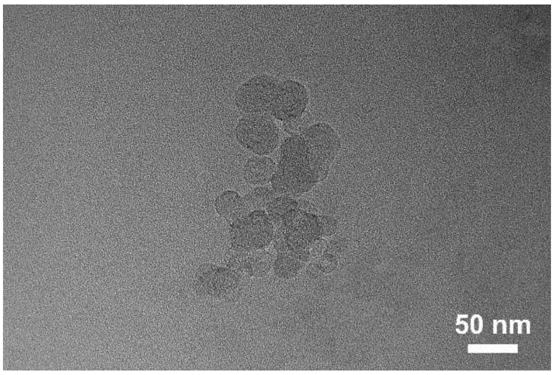

Supplementary Figure 22 | Morphology of the p-HC-G4D SADs from HRTEM images.

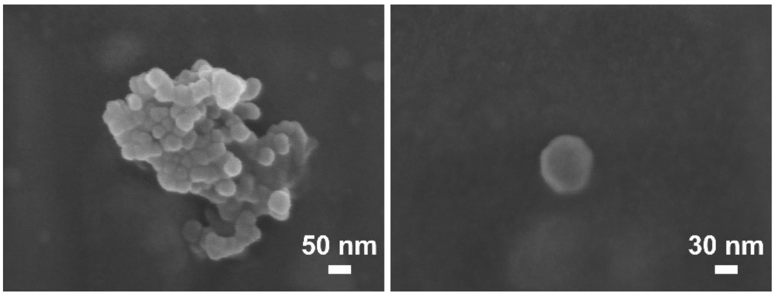

Supplementary Figure 23 | Morphology of the p-HC-G4D SADs from SEM images.

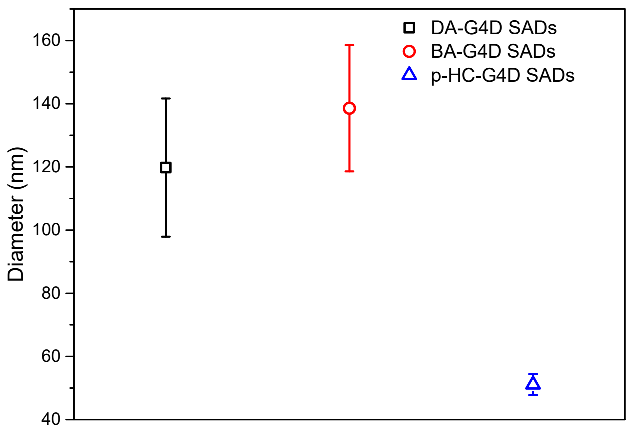

Supplementary Figure 24 | Diameter of the three types of SADs from SEM images.

The diameter of the DA-G4D, BA-G4D and p-HC-G4D SADs from SEM images is 119.8 ± 21.88 nm, 138.57 ± 20 nm and 51.1 ± 3.33 nm, respectively. The error bars represent the average size data obtained from the measurement of the SEM images of SADs.

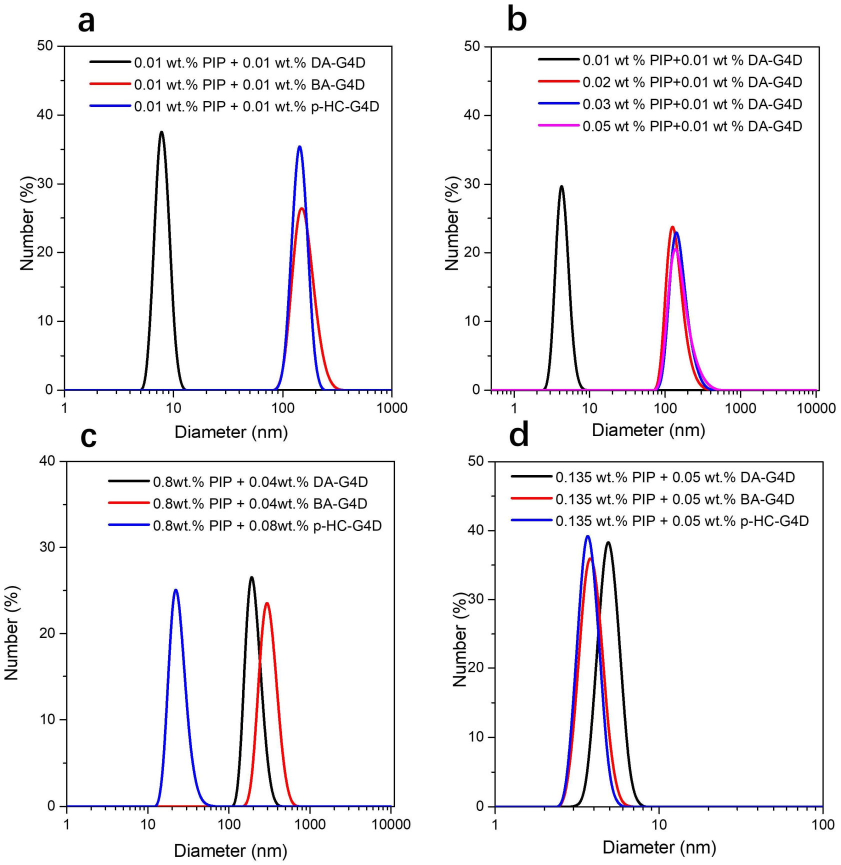

Supplementary Figure 25 | a Nanoparticle size distribution of the self-assembled dendrimers (SADs) in lower PIP and dendrimer concentrations, b Nanoparticle size distribution of the DA-G4D self-assembled dendrimers (SADs) in different PIP concentrations, c Nanoparticle size distribution of the self-assembled dendrimers (SADs) in optimized PIP and dendrimer concentration, d Nanoparticle size distribution of the self-assembled dendrimers (SADs) in 0.8 wt.% PIP and 1 wt.% dendrimer concentration when diluted 20 times with 0.1 wt.% PIP concentration. The final concentrations calculated for PIP and dendrimer are 0.135 wt.% and 0.05 wt.%, respectively.

As shown in Supplementary Figure 25a, under the low PIP and dendrimer concentrations, BA-G4D and p-HC-G4D show the larger size distributions than that of the DA-G4D. And when the PIP concentration was increased up to 0.02wt.%, 0.03 wt.% and 0.05 wt.%, sizes of the DA-G4D self-assembled dendrimers (SADs) were turned into large accordingly (Supplementary Figure 25b). Moreover, nanoparticle size distribution of the self-assembled dendrimers (SADs) in 0.8 wt.% PIP and 1 wt.% dendrimer concentration was decreased to less than 10 nm when diluted 20 times with 0.1 wt.% PIP concentration (Supplementary Figure 25d).

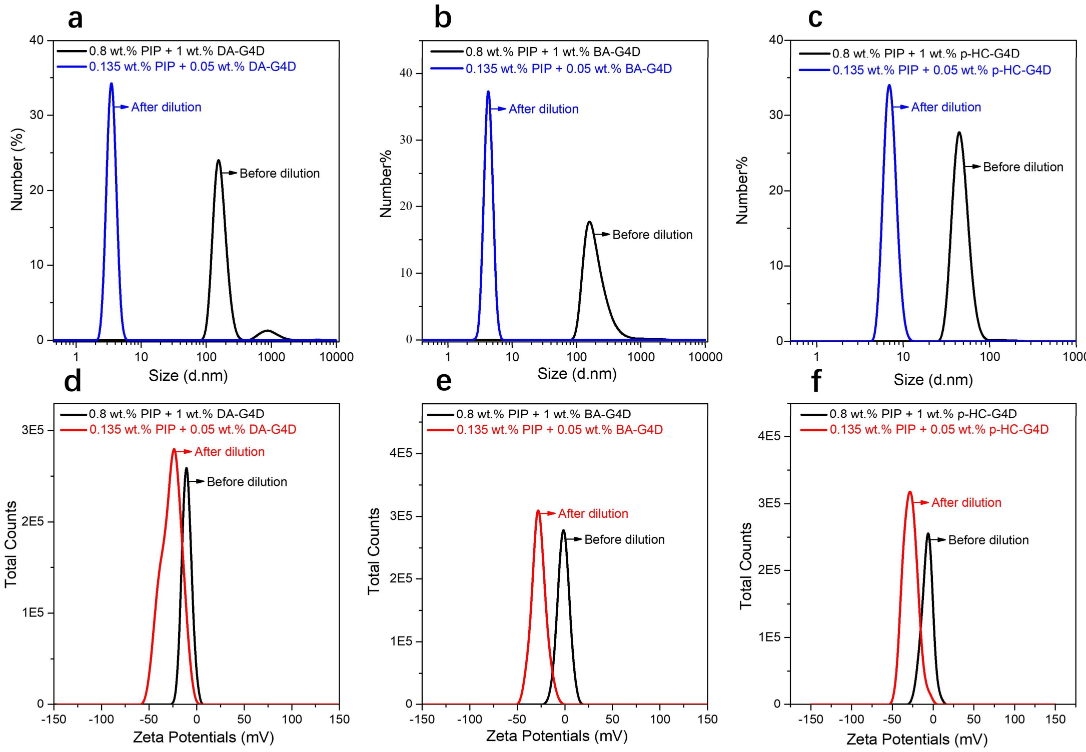

Supplementary Figure 26 | Size distribution (a, b, c) and zeta potentials (d, e, f) of the

self-assemble dendrimers (composition: 0.8 wt.% PIP and 1 wt.% dendrimer) before and after dilution using 0.1 wt.% PIP concentration.

We investigated the size distribution and zeta potentials of the self-assemble dendrimers before and after dilution using 0.1 wt.% PIP concentration. As shown in Figs. 26a–26c, when using 0.1 wt.% PIP to dilute the 0.8 wt.% and 1 wt.% dendrimer (SADs), these self-assemble dendrimers (SADs) became the single dendrimer nanoparticle, which was consistent with Supplementary Figure 25d. Zeta potentials data in Figs. 26d–26f further illustrate that, these self-assemble dendrimers (SADs) before dilution basically all showed a neutral charge surface, while after dilution, their surfaces turned into negative charges. These results demonstrate that using 0.1 wt.% PIP to dilute the SADs solution (containing 0.8 wt.% PIP and 1 wt.% dendrimer) can alter the electrostatic interaction between dendrimers and PIP molecules, further result in the disaggregation of the SADs formed, then finally turn into the single dendrimer nanoparticle.

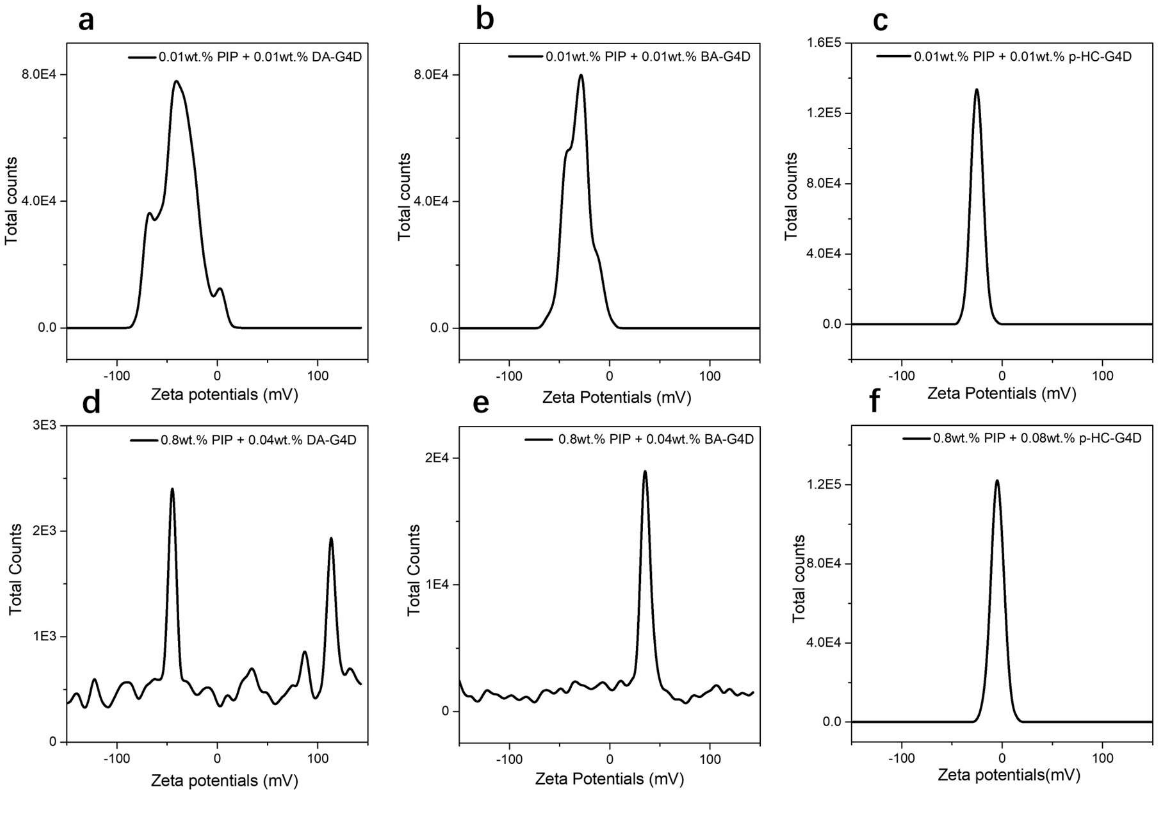

Supplementary Figure 27 | Zeta potential of the self-assembled dendrimers (SADs) in

different PIP and dendrimer concentration: a 0.01 wt.% PIP + 0.01 wt.% DA-G4D, b 0.01 wt.% PIP + 0.01 wt.% BA-G4D, c 0.01 wt.% PIP + 0.01 wt.% p-HC-G4D, d 0.8 wt.% PIP + 0.04 wt.%DA-G4D, e 0.8 wt.% PIP + 0.04 wt.% BA-G4D, f 0.8 wt.% PIP + 0.04 wt.% p-HC-G4D.

As shown in Supplementary Figures 27d–27f, under the optimized PIP and dendrimer concentration for the preparation of PA nanofilms, the formed SADs exhibit a positive charge, demonstrating that the peripheral of the SADs possess lots of PIP molecules. However, when the PIP and dendrimer concentrations are reduced to 0.01 wt.% and 0.01 wt.%, respectively, the formed SADs and partial dendrimers dissolved in PIP solution show negative charge, which means the outsides of these nanoparticles have not enough PIP molecules, thus showing the charge of carboxylic acid group and phenolic hydroxyl group (Supplementary Figures 27a–27c). For example, the BA-G4D SADs show a positive charge of 35.45 mV in the optimized PIP and BA-G4D concentrations, whereas the BA-G4D dendrimer show a negative charge of –28.37 mV in the 0.01 wt.% PIP + 0.01 wt.% BA-G4D. Hence, this result gives the confirmation that the peripheries of SADs are featured with aggregated PIP molecules and can be involved in the IP to form amide bond, and fine-tune the nanofilm inner structure.

2.3 Structural characterization of the resulted membranes

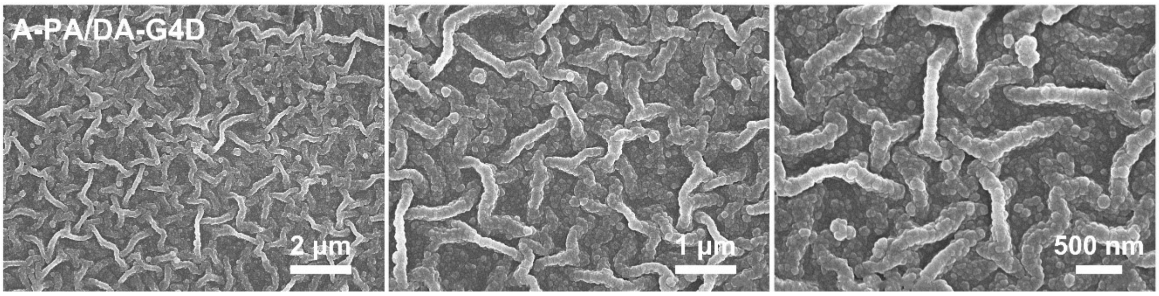

Supplementary Figure 28 | Surface morphology SEM images of the A-PA/DA-G4D nanofilm formed on the PSF support with dendrimer porous layer. Fabrication condition: reaction time 30s.

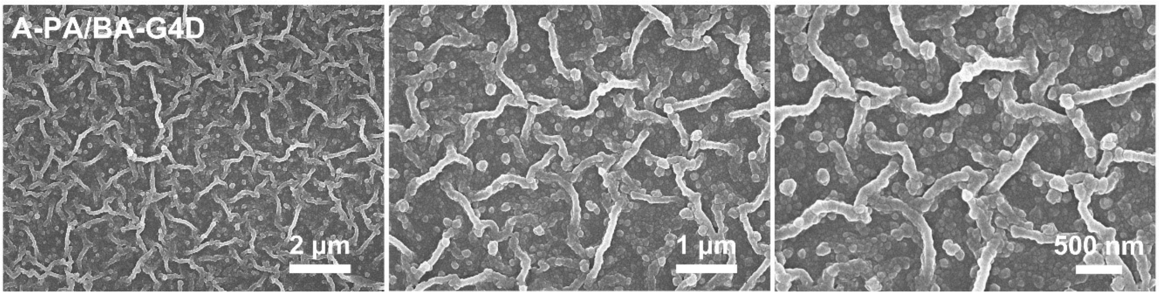

Supplementary Figure 29 | Surface morphology SEM images of the A-PA/BA-G4D nanofilm formed on the PSF support with dendrimer porous layer. Fabrication condition: reaction time 30s.

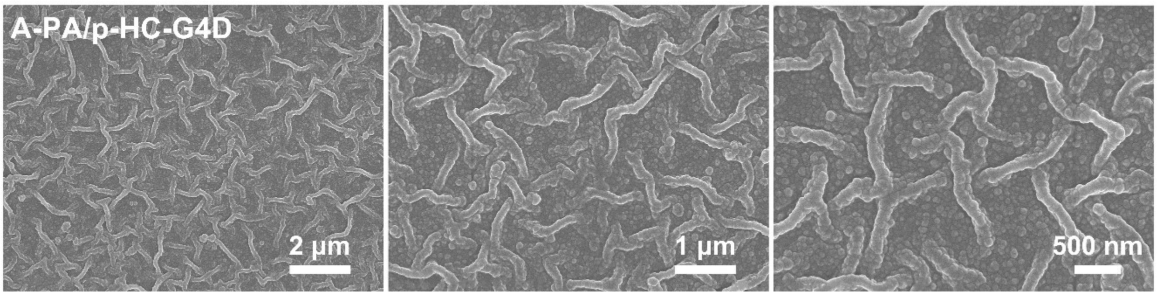

Supplementary Figure 30 | Surface morphology SEM images of the A-PA/P-HC-G4D nanofilm formed on the PSF support with dendrimer porous layer. Fabrication condition: reaction time 30s.

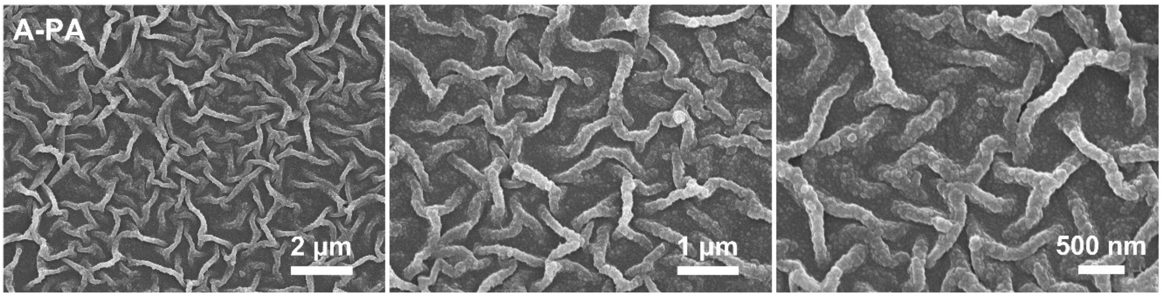

Supplementary Figure 31 | Surface morphology SEM images of the A-PA nanofilm formed on the PSF support with dendrimer porous layer. Fabrication condition: reaction time 30s.

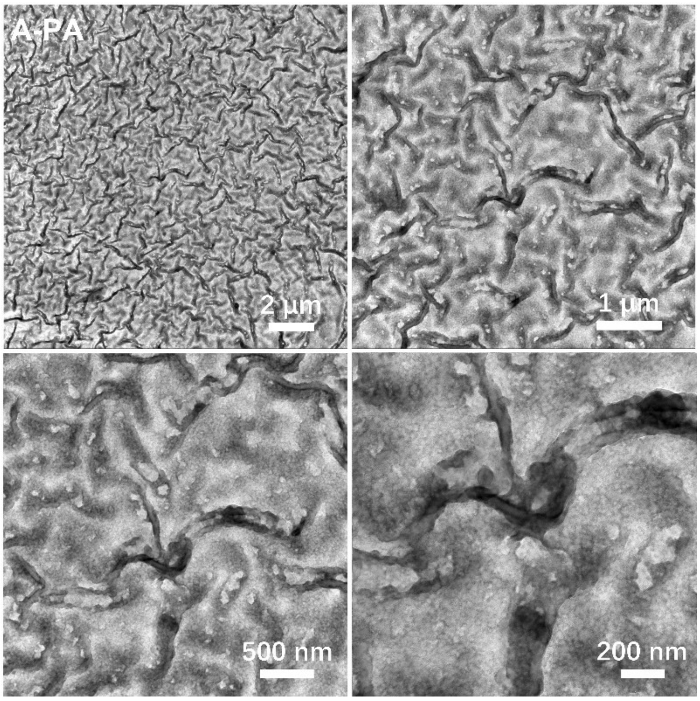

Supplementary Figure 32 | Bottom to top TEM micrographs of the A-PA nanofilm formed on the PSF support with dendrimer porous layer. Fabrication condition: reaction time 30s.

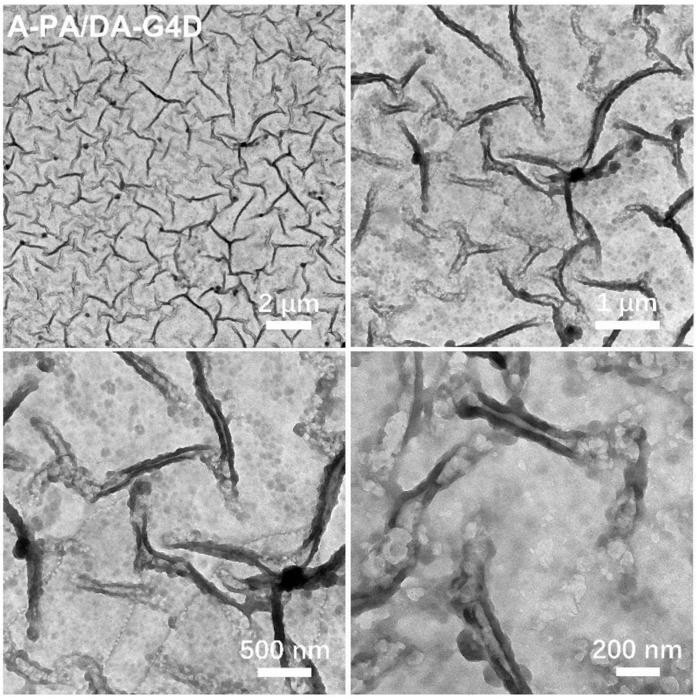

Supplementary Figure 33 | Bottom to top TEM micrographs of the A-PA/DA-G4D nanofilm formed on the PSF support with dendrimer porous layer. Fabrication condition: reaction time 30s.

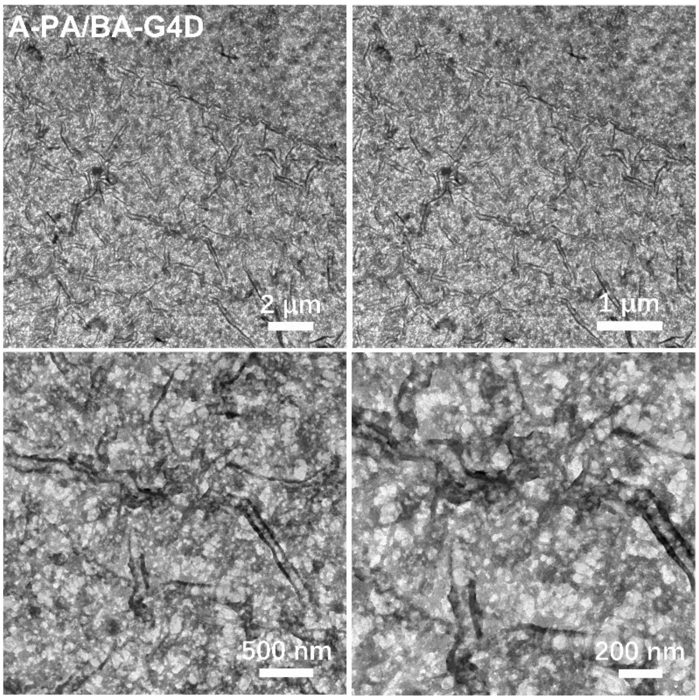

Supplementary Figure 34 | Bottom to top TEM micrographs of the A-PA/BA-G4D nanofilm formed on the PSF support with dendrimer porous layer. Fabrication condition: reaction time 30s.

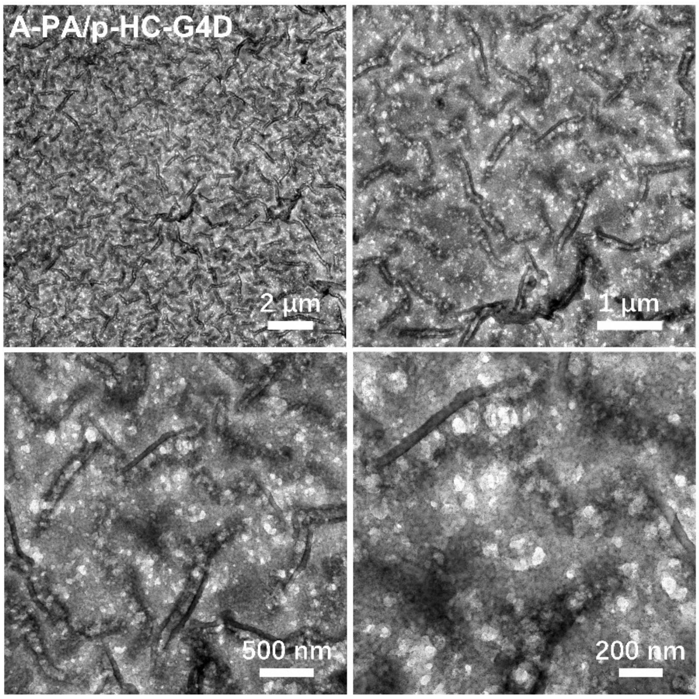

Supplementary Figure 35 | Bottom to top TEM micrographs of the A-PA/p-HC-G4D nanofilm formed on the PSF support with dendrimer porous layer. Fabrication condition: reaction time 30s.

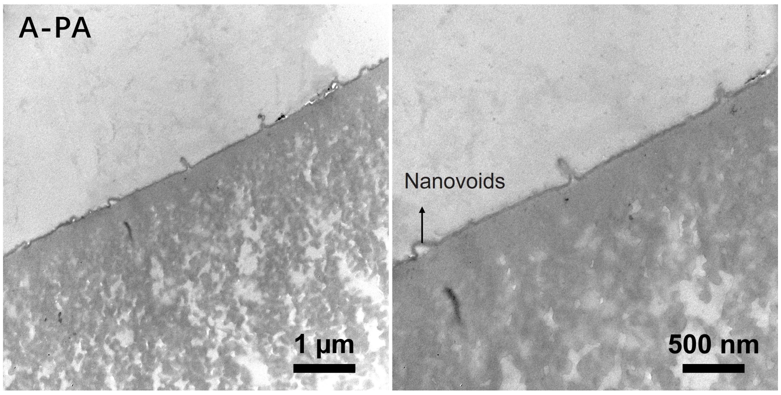

Supplementary Figure 36 | Cross-sectional morphology of A-PA membrane characterized by TEM.

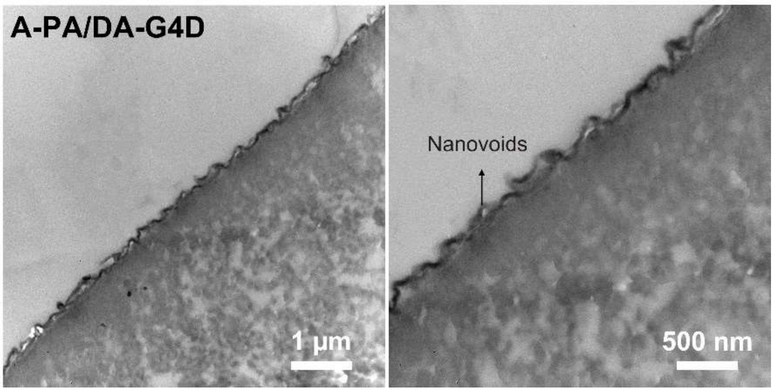

Supplementary Figure 37 | Cross-sectional morphology of A-PA/DA-G4D membrane characterized by TEM.

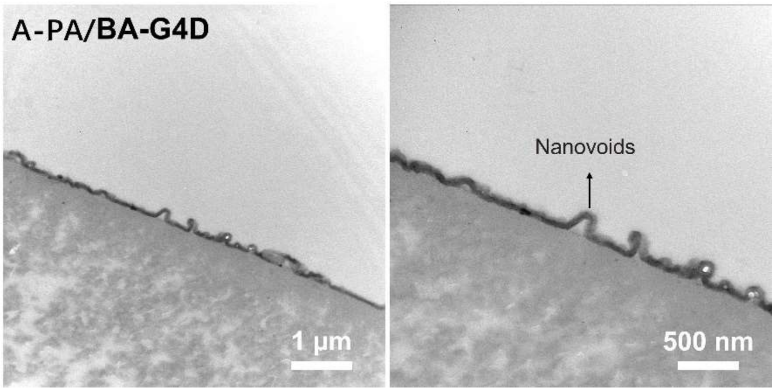

Supplementary Figure 38 | Cross-sectional morphology of A-PA/BA-G4D membrane characterized by TEM.

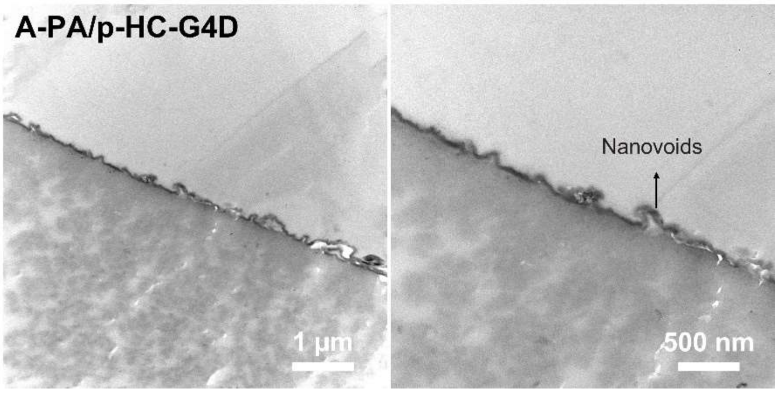

Supplementary Figure 39 | Cross-sectional morphology of A-PA/p-HC-G4D membrane characterized by TEM.

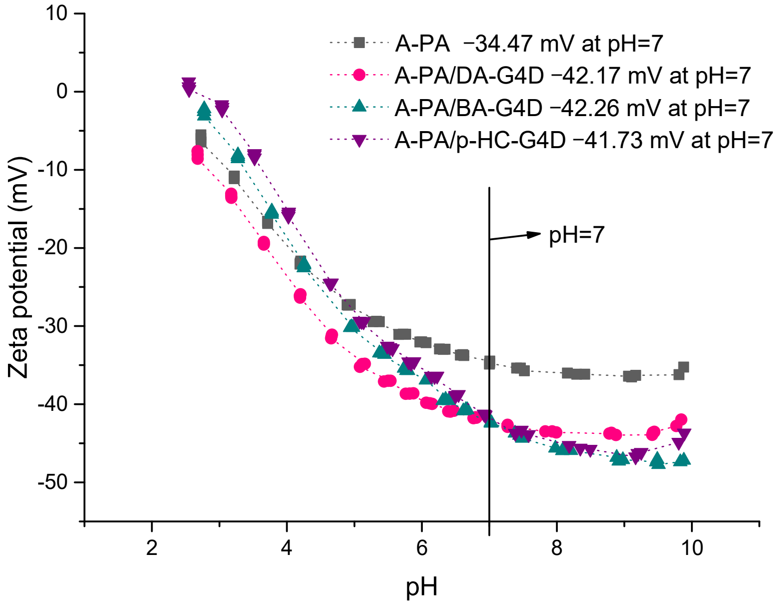

Supplementary Figure 40 | Representative ζ potential of the A-PA, A-PA/DA-G4D, A-PA/BA-G4D and A-PA/p-HC-G4D membranes. The membrane zeta potentials were estimated by measuring the ζ potentials in a background electrolyte solution of 1 mM KCl. All measurements were performed at 25°C and repeated 4 times.

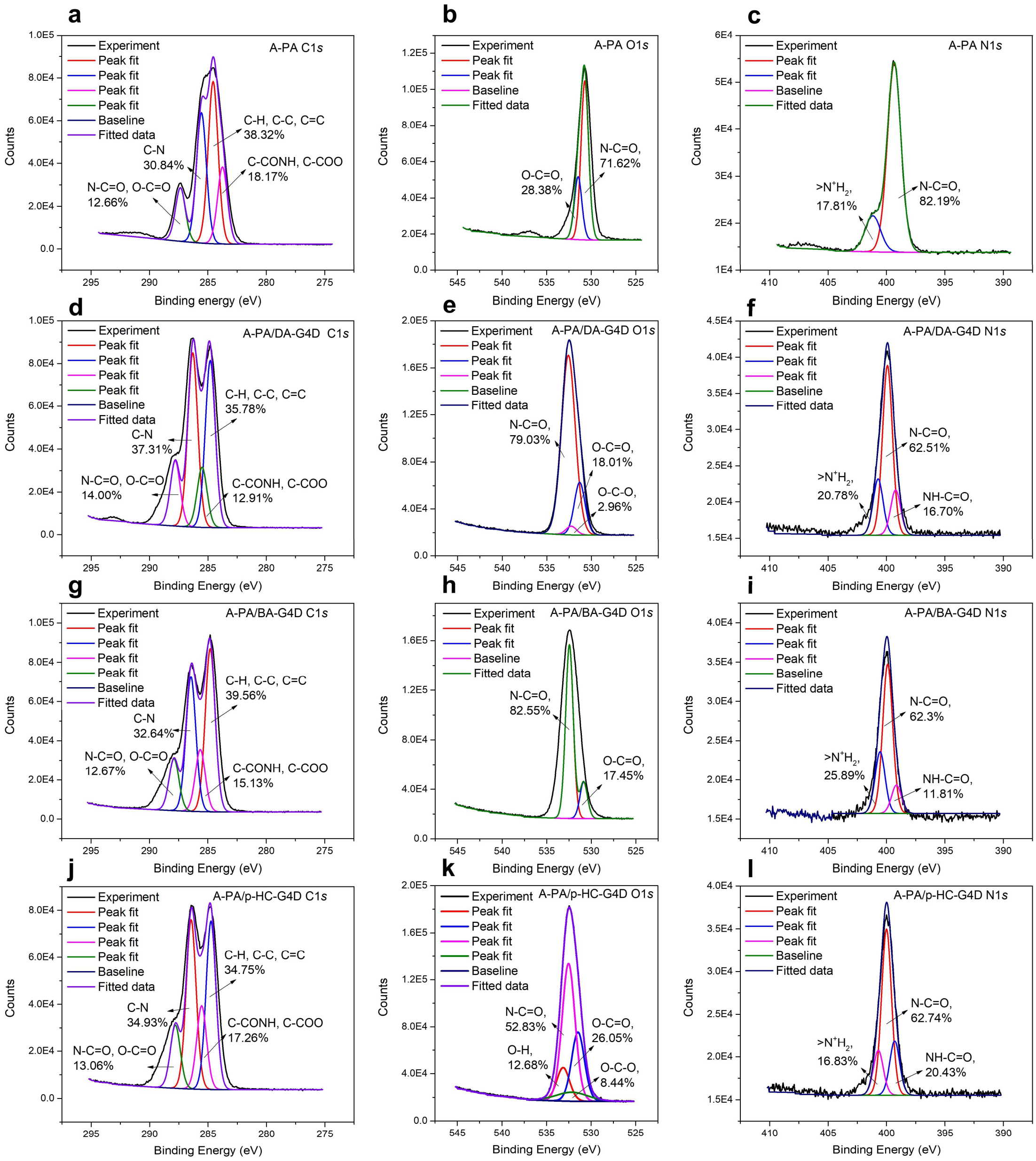

Supplementary Figure 41 | a, b, c C1*s*, O1*s* and N1*s* Scan results of the X-ray photoelectron spectra of the A-PA nanofilm. d, e, f C1*s*, O1*s* and N1*s* Scan results of the X-ray photoelectron spectra of the A-PA/DA-G4D nanofilm. g, h, i C1*s*, O1*s* and N1*s* Scan results of the X-ray photoelectron spectra of the A-PA/BA-G4D nanofilm. j, k, l C1*s*, O1*s* and N1*s* Scan results of the X-ray photoelectron spectra of the A-PA/p-HC-G4D nanofilm.

As Supplementary Figure 41, O1*s* and N1*s* scan results of the X-ray photoelectron spectra show that the SADs were incorporated into the PA layer. To be specific, take the A-PA/p-HC-G4D nanofilm as an example, the O1*s* XPS spectra of the resulted nanofilms show the –O–C–O and –O–H fitted peaks, as relative to the pristine A-PA

nanofilm. Moreover, the N1*s* XPS spectra of the A-PA/p-HC-G4D nanofilm show the existence of NH–C=O fitted peaks as a result of the addition of the p-HC-G4D. Similar results also were found in the A-PA/DA-G4D and A-PA/BA-G4D nanofilms.

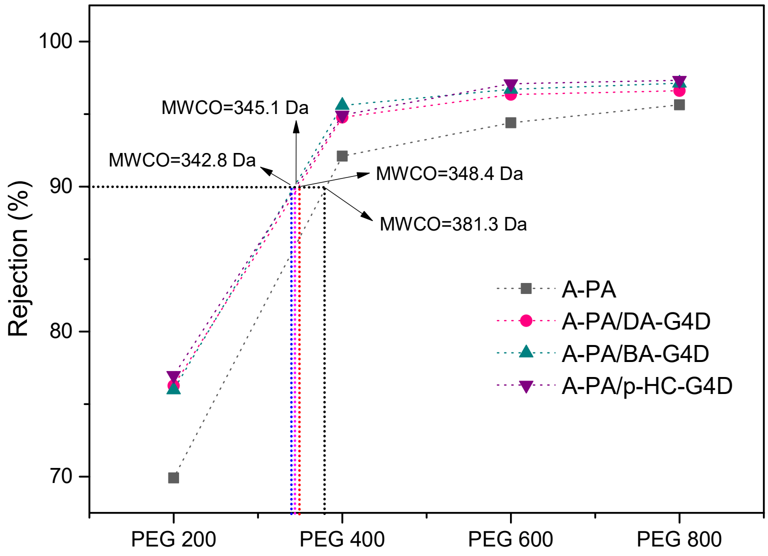

Supplementary Figure 42 | Rejection curves to PEG with different molecular weight.

As for the effective pore size, the molecular weight cut-off (MWCO) of the traditional PA and SADs polyamide membranes were determined through permeation tests to the PEG with different molecular weight, such as 200 Da, 400 Da, 600 Da, and 800 Da. As shown in Supplementary Figure 42, the MWCOs for the A-PA, A-PA/DA-G4D, A-PA/BA-G4D and A-PA/p-HC-G4D membranes are 381.3 Da, 348.4 Da, 342.8 Da, and 345.1 Da, respectively, which corresponds to the effective pore radius of about 2.136 Å, 1.827 Å, 1.888 Å and 1.887 Å. These results indicate that incorporating SADs into PA nanofilms can narrow mean effective pore size.

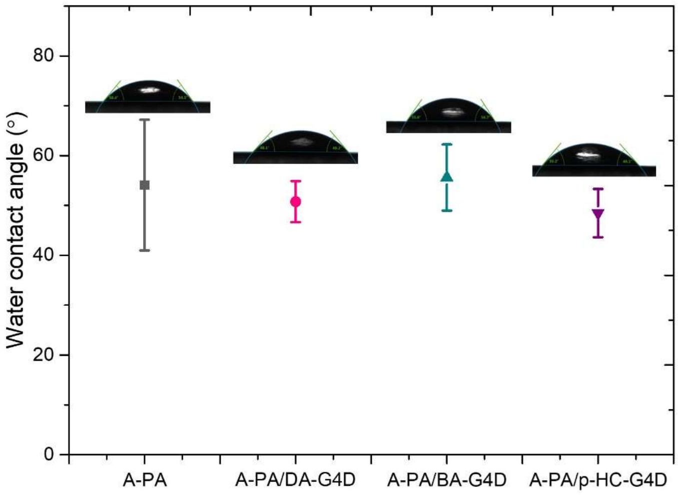

Supplementary Figure 43 | Water contact angle (CA) on the surface of the A-PA, A-PA/DA-G4D, A-PA/BA-G4D and A-PA/p-HC-G4D nanofilms. The error bars represent the reproducible water contact angle data obtained from at least three independent membrane samples.

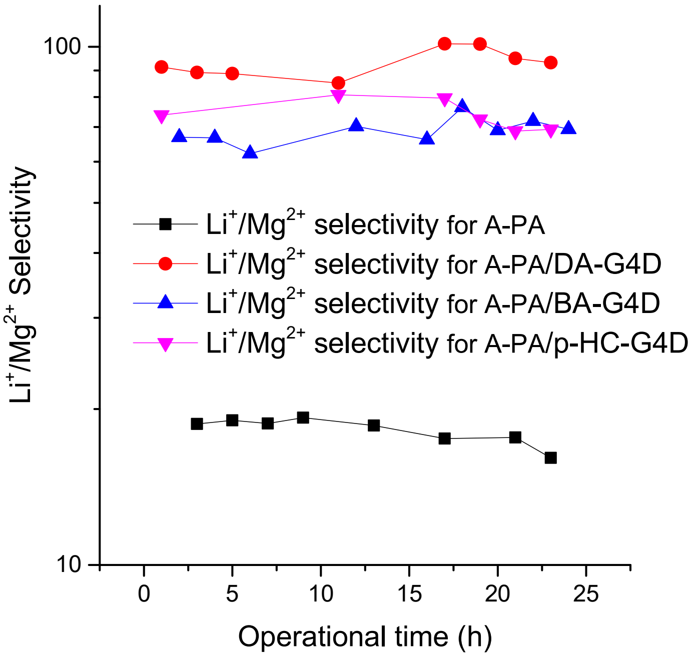

Supplementary Figure 44 | Li^+/ Mg^{2+} selectivity operational stability of the fabricated polyamide membranes.

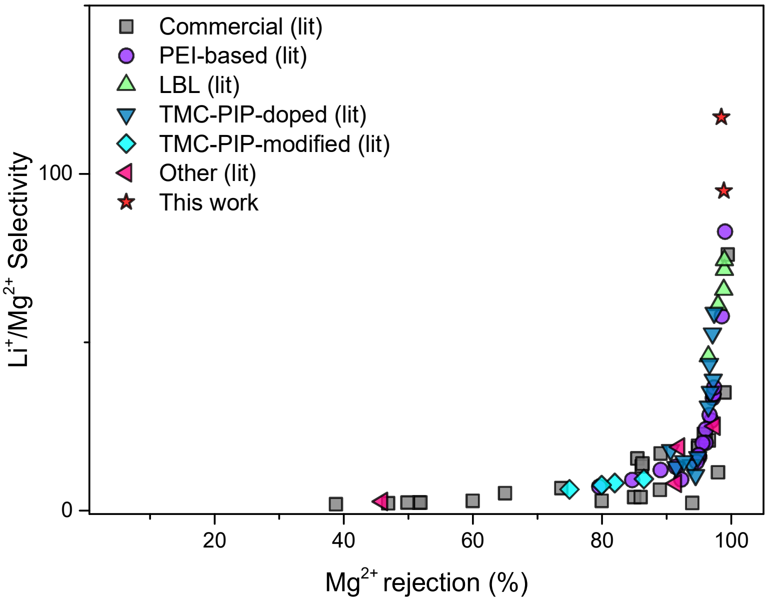

Supplementary Figure 45 | Li^{+}/ Mg^{2+} selectivity as a function of Mg^{2+} rejection.

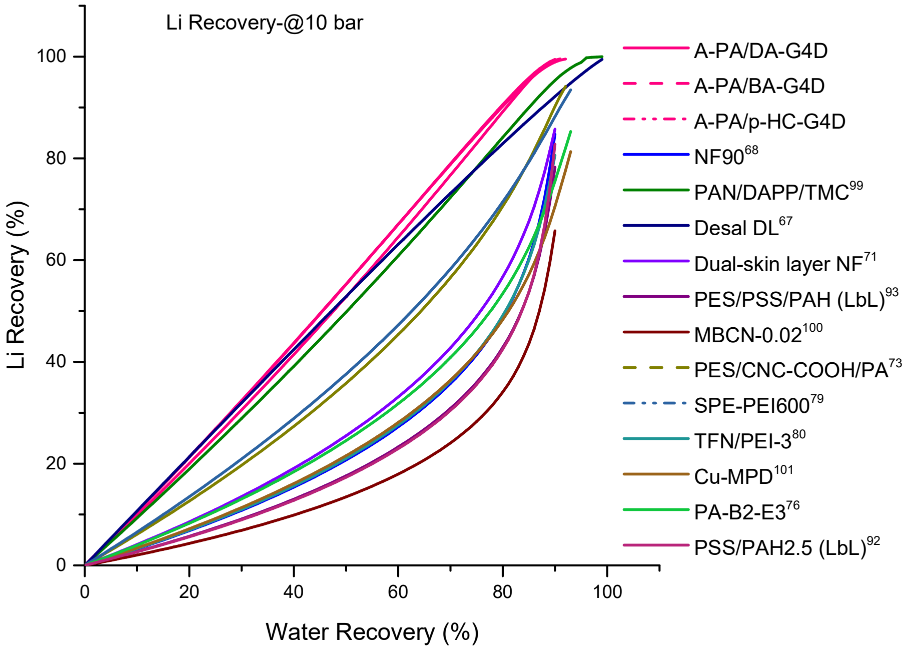

Supplementary Figure 46 | Module analysis: Calculated Li^{+} recovery vs water recovery along a simulated module. Feed conditions: Li^{+} concentration of 3.4 mM, Mg^{2+} of 19.6 mM, and operating pressure of 10 bar (1 MPa). Membrane properties: choosing the best-reported data for each type of membrane.

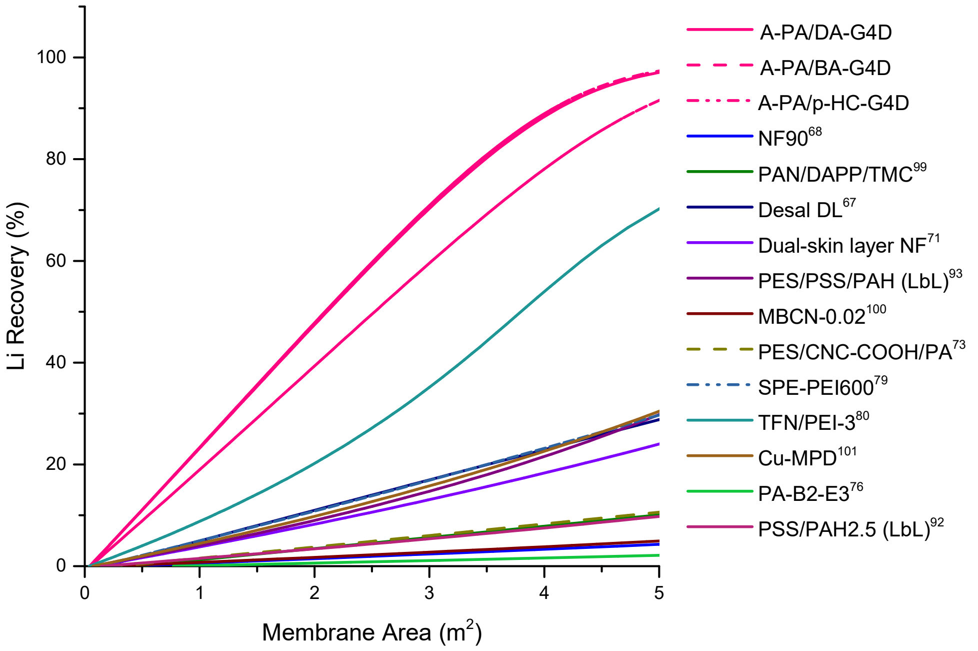

Supplementary Figure 47 | Module analysis: Calculated Li^+ recovery vs membrane area along a simulated module. Feed conditions: Li^+ concentration of 3.4 mM, Mg^{2+} of 19.6 mM, and operating pressure of 10 bar (1 MPa). Membrane properties: choosing the best-reported data for each type of membrane.

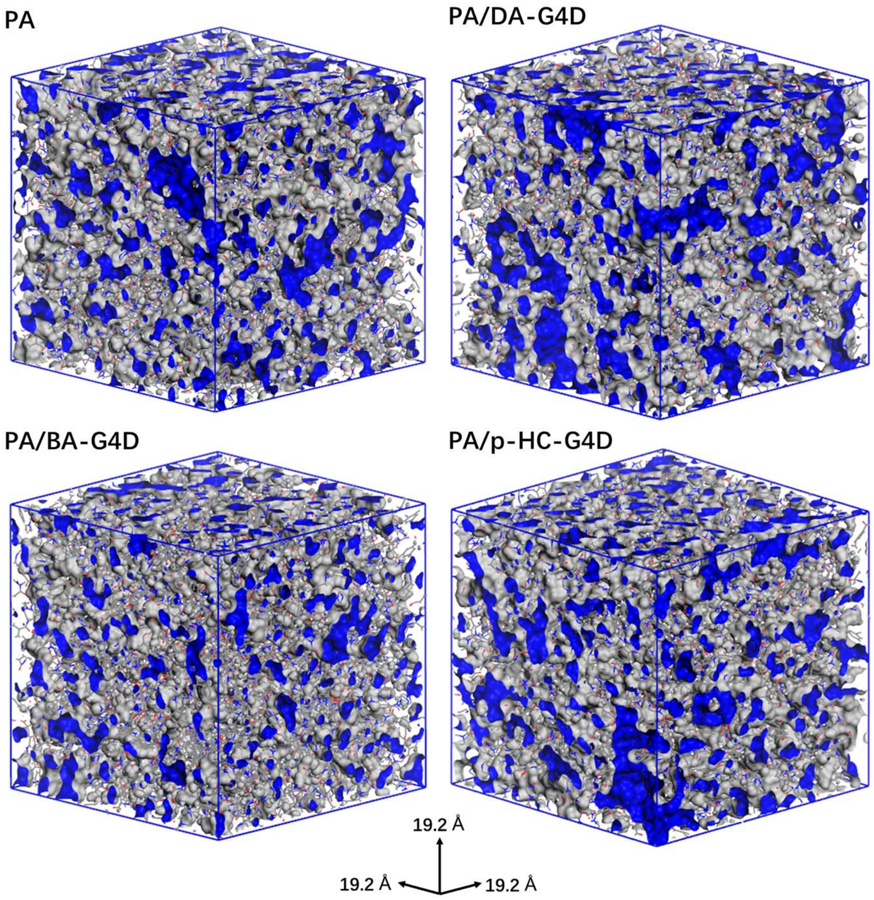

Supplementary Figure 48 | Three-dimensional views of four amorphous cells, each containing a distinct polyamide network, of which PA/DA-G4D, PA/BA-G4D and PA/p-HC-G4D are polyamide network containing different dendrimers. Visualisation model carried out in Materials Studio. Blue colour: surface at probe radius of 1 Å diameter. Cell size: 96 × 96 × 96 Å. The boxes show that there are more voids and that there is greater connectivity between voids for the PA/DA-G4D, PA/BA-G4D and PA/p-HC-G4D polymer networks than for the PA polymer networks.

3 Supplementary Tables

Supplementary Table 1 | Cl^-/SO_4^{2-} separation selectivity ($\alpha_{Cl^-/SO_4^{2-}}$) of the A-PA, A-PA/DA-G4D, A-PA/BA-G4D, A-PA/p-HC-G4D and commercial PA membranes under a feed solution of 2 g L^{-1} NaCl and 2 g L^{-1} Na_2SO_4. Test condition: 1 MPa, 25°C, 7.5 LPM.

Membrane	Ion rejection rate (%)		$\alpha_{Cl^-/SO_4^{2-}}$
	Cl^-	SO_4^{2-}	
A-PA	14.34	99.49	167.96
A-PA/DA-G4D	14.85	99.70	283.83
A-PA/BA-G4D	42.34	99.98	2883
A-PA/p-HC-G4D	–48.73	99.91	1652.56
NF 270	13.1	98.9	79
XC-N	14.32	98.8	71.4
DK	–13.88	98.8	94.9
DL	–3.09	98.9	88.1

Supplementary Table 2 | Cl^-/SO_4^{2-} separation selectivity ($\alpha_{Cl^-/SO_4^{2-}}$) from the literature reported membranes. Noted that the test conditions are using the mixed solutions.

Membrane	Test conditions (Mixed solutions)	$\alpha_{Cl^-/SO_4^{2-}}$	Water flux permeance (L/m^2 h MPa)	Ref.
H40/PIP	0.4 MPa<br>1.0 g · L^{-1} NaCl and 1.0 g · L^{-1} Na_2SO_4	120	162.5 to 2.0 g · L^{-1} Na_2SO_4	5
H40/PIP	0.4 MPa<br>2.0 g L^{-1} NaCl and 2.0 g L^{-1} Na_2SO_4	59.6	162.5 to 2.0 g · L^{-1} Na_2SO_4	5
PIP only	0.4 MPa<br>1.0 g L^{-1} NaCl and 1.0 g L^{-1} Na_2SO_4	43.6	109.1 to 2.0 g · L^{-1} Na_2SO_4	5
PIP only	0.4 MPa<br>2.0 g L^{-1} NaCl and 2.0 g L^{-1} Na_2SO_4	24.5	109.1 to 2.0 g · L^{-1} Na_2SO_4	5
A-CPTC-PIP	1 MPa<br>1.0 g L^{-1} NaCl and 1.0 g L^{-1} Na_2SO_4	76.8	133.4 to 2.0 g · L^{-1} Na_2SO_4	6
A-CPTC-PIP	1 MPa<br>2.0 g L^{-1} NaCl and 2.0 g L^{-1} Na_2SO_4	98.4	133.4 to 2.0 g · L^{-1} Na_2SO_4	6
DK	1 MPa<br>1.0 g L^{-1} NaCl and 1.0 g L^{-1} Na_2SO_4	35.6	68.4 to 2.0 g · L^{-1} Na_2SO_4	6

DK	1 MPa 2.0 g L^{-1} NaCl and 2.0 g L^{-1} Na_2SO_4	65.6	68.4 to 2.0 g·L^{-1} Na_2SO_4	6
DL	1 MPa 1.0 g L^{-1} NaCl and 1.0 g L^{-1} Na_2SO_4	37.2	47.3 to 2.0 g·L^{-1} Na_2SO_4	6
DL	1 MPa 2.0 g L^{-1} NaCl and 2.0 g L^{-1} Na_2SO_4	72.2	47.3 to 2.0 g·L^{-1} Na_2SO_4	6
NF 270	1 MPa 1.0 g L^{-1} NaCl and 1.0 g L^{-1} Na_2SO_4	37.8	107.4 to 2.0 g·L^{-1} Na_2SO_4	6
NF270	1 MPa 2.0 g L^{-1} NaCl and 2.0 g L^{-1} Na_2SO_4	69.4	107.4 to 2.0 g·L^{-1} Na_2SO_4	6
XC-N	1 MPa 1.0 g L^{-1} NaCl and 1.0 g L^{-1} Na_2SO_4	34.7	89.7 to 2.0 g·L^{-1} Na_2SO_4	6
XC-N	1 MPa 2.0 g L^{-1} NaCl and 2.0 g L^{-1} Na_2SO_4	57.9	89.7 to 2.0 g·L^{-1} Na_2SO_4	6
Pre-diffusion IP	0.4 MPa 250 ppm Cl^-, and 250 ppm SO_4^{2-}	246	64 to 250 ppm Cl^- and 250 ppm SO_4^{2-}	7
Surfactant-assembly regulated IP	0.4 MPa 2.0 g L^{-1} NaCl and 2.0 g L^{-1} Na_2SO_4	182.5	171 to pure water	8
PDA/CNT interlayered TFC	—	85	210	9
Zwitterion CNT membranes	—	93	188	10
Titanate nanotubes TFN membranes	—	22.5	74.8	11
Oligo-ethylene-glycol TFC	0.6 MPa, 2 g L^{-1} of mixed feed solution	85	86	12
NF1	1.0 g L^{-1} mixed NaCl/Na_2SO_4 aqueous solution at the weight ratio of 1:1 under 0.5 MPa	23.1	148 to pure water	13
NF2	1.0 g L^{-1} mixed NaCl/Na_2SO_4 aqueous solution at the weight	25.2	164 to pure water	13

	ratio of 1:1 under 0.5 MPa			
NFMs@SMPS 12h	Na_2SO_4/NaCl mass ratio = 2:1 at concentration of 1.0 g L^{-1}	23.6	33	[14]
NF-0.1%-CB-1	0.6 MPa<br>Mixed solution of 1.0 g L^{-1} Cl^- or 1.0 g L^{-1} SO_4^{2-}	16	155	[15]
PIP-TMC-QAEP	0.6 MPa<br>Mixture solution of NaCl and Na_2SO_4<br>(Overall concentration: 2 g L^{-1} ~ 10 g L^{-1}	38.1	185	[16]
PIP-TMC	0.6 MPa<br>Mixture solution of NaCl and Na_2SO_4<br>(Overall concentration: 2 g L^{-1} ~ 10 g L^{-1}	36.4	62	[16]
TFC NFMs with alginate selective layers	0.6 MPa<br>1.7 g L^{-1} NaCl or 3.0 g·L^{-1} Na_2SO_4	36.4	131	[17]
charged PA nanofilm	0.4 MPa<br>0.5 g L^{-1} NaCl/0.5 g L^{-1} Na_2SO_4	>65	103.5 to pure water	[18]
SCOF/PA	0.5 MPa<br>0.5 g L^{-1} NaCl/0.5 g L^{-1} Na_2SO_4	312.6	122 to pure water	[19]
ZIF-8/PSF-1	1 MPa<br>1.0 g L^{-1} NaCl and 1.0 g L^{-1} Na_2SO_4	200.6	167.64 to 2.0 g·L^{-1} Na_2SO_4	[20]
ZIF-8/PSF-1	1 MPa<br>2.0 g L^{-1} NaCl and 2.0 g L^{-1} Na_2SO_4	377.8	167.64 to 2.0 g·L^{-1} Na_2SO_4	[20]
N-TFN	0.4 MPa<br>4 g L^{-1} NaCl/2 g L^{-1} Na_2SO_4	50	417 to 1.0 g·L^{-1} Na_2SO_4	[21]

Supplementary Table 3 | Membrane performance data are taken from the literature to calculate the single salt selectivity [NaCl to Na_2SO_4] of the nanofiltration membranes. Noted that the test conditions are using the single salt solution.

Membrane	Test conditions (Single solutions)	$\alpha_{Cl^-/SO_4^{2-}}$	Water permeance (L/m^2 h MPa)	Ref.
NFM-15	1 MPa 2.0 g L^{-1} NaCl or 2.0 g L^{-1} Na_2SO_4	114	237	22
PIP–CSP6/TMC	0.5 MPa 1.0 g L^{-1} NaCl or 1.0 g L^{-1} Na_2SO_4	106	452	23
THPC-5	0.6 MPa 1.0 g L^{-1} NaCl or 1.0 g L^{-1} Na_2SO_4	48.8	505	24
PA20/PAN TFNC	0.5 MPa 2.0 g L^{-1} NaCl or 2.0 g L^{-1} Na_2SO_4	82	258	25
PA@W-14	0.5 MPa 2.0 g L^{-1} NaCl or 2.0 g L^{-1} Na_2SO_4	44.8	265	26
PES-COFs scaffold/PIPTMC polyamide	0.4 MPa 1.0 g L^{-1} NaCl or 1.0 g L^{-1} Na_2SO_4	17.6	311	27
PA/M-50 (PIP 0.05/TMC 0.05)	0.6 MPa ——	36	262	28
TFC-SDS	0.6 MPa 1.0 g L^{-1} NaCl or 1.0 g L^{-1} Na_2SO_4	6.9	75	29
M-U4-O	0.6 MPa 2.0 g L^{-1} NaCl or 2.0 g L^{-1} Na_2SO_4	170.7	79	30
PIP-0.8-60	0.4 MPa 1.0 g L^{-1} NaCl or 1.0 g L^{-1} Na_2SO_4	76.6	220	31
PIP-0.3-60	0.4 MPa 1.0 g L^{-1} NaCl or 1.0 g L^{-1} Na_2SO_4	19.5	347	31
PIP 0.0175 wt%	0.4 MPa 1.0 g L^{-1} NaCl or 1.0 g L^{-1} Na_2SO_4	23.0	528	32
PIP 0.015 wt%	0.4 MPa 1.0 g L^{-1} NaCl or 1.0 g L^{-1} Na_2SO_4	13.8	629	32
PA/CLS(5)	0.2 MPa 1.0 g L^{-1} NaCl or 1.0 g L^{-1} Na_2SO_4	12.7	535	33
TPT-TMC/PSf TFC	0.69 MPa 2.0 g L^{-1} NaCl or 2.0 g L^{-1} Na_2SO_4	42.5	93	34
PIP-TMC/PSf TFC	0.69 MPa 2.0 g L^{-1} NaCl or 2.0 g L^{-1} Na_2SO_4	19.1	82	34

PA-TFC	0.6 MPa<br>5 mmol NaCl or<br>5 mmol Na_2SO_4	15.5	30	35
TFN-SCQD	0.6 MPa<br>5 mmol NaCl or<br>5 mmol Na_2SO_4	14.7	70	35
TFN-NCQD	0.6 MPa<br>5 mmol NaCl or<br>5 mmol Na_2SO_4	8.4	52	35
TFN-CCQD	0.6 MPa<br>5 mmol NaCl or 5 mmol Na_2SO_4	13.0	61	35
M10-c membrane	0.2 MPa<br>0.5 g L^{-1} NaCl or 0.5 g L^{-1} Na_2SO_4	10.4	123	36
M2-c membrane	0.2 MPa<br>0.5 g L^{-1} NaCl or 0.5 g L^{-1} Na_2SO_4	13.4	136	36
PMIA-TFC	0.6 MPa<br>1.0 g L^{-1} NaCl or 1.0 g L^{-1} Na_2SO_4	4.3	16	37
TFC0	0.345 MPa<br>1.0 g L^{-1} NaCl or 1.0 g L^{-1} Na_2SO_4	3.7	22	38
TFCn	0.345 MPa<br>1.0 g L^{-1} NaCl or 1.0 g L^{-1} Na_2SO_4	17.2	197	38
TFNM with HZNCs	0.6 MPa<br>1.0 g L^{-1} NaCl or 1.0 g L^{-1} Na_2SO_4	11.7	165	39
TFNM with ZNPs	0.6 MPa<br>1.0 g L^{-1} NaCl or 1.0 g L^{-1} Na_2SO_4	8.7	145	39
Control TFC NF	0.6 MPa<br>1.0 g L^{-1} NaCl or 1.0 g L^{-1} Na_2SO_4	8.2	105	39
H-TFC	0.6 MPa<br>1.0 g L^{-1} NaCl or 1.0 g L^{-1} Na_2SO_4	54.8	130	40
TFC-R	0.6 MPa<br>2.0 g L^{-1} NaCl or 2.0 g L^{-1} Na_2SO_4	94.2	213	41
TFC-T	0.6 MPa<br>2.0 g L^{-1} NaCl or 2.0 g L^{-1} Na_2SO_4	34.5	57	41
HNTs	0.4 MPa<br>1.0 g L^{-1} NaCl or 1.0 g L^{-1} Na_2SO_4	39.8	345	42
PA/GE20/PAN	0.4 MPa<br>1.0 g L^{-1} NaCl or 1.0 g L^{-1} Na_2SO_4	45.1	357	43
SDA 2%	1 MPa<br>2.0 g L^{-1} NaCl or 2.0 g L^{-1} Na_2SO_4	3.3	62	44
SDA/PIP	1 MPa<br>2.0 g L^{-1} NaCl or 2.0 g L^{-1} Na_2SO_4	7.3	50	44
CDA 2%	1 MPa<br>2.0 g L^{-1} NaCl or 2.0 g L^{-1} Na_2SO_4	3	53	44

CDA/PIP	1 MPa<br>2.0 g L^{-1} NaCl or 2.0 g L^{-1} Na_2SO_4	4.1	42	44
PIP	1 MPa<br>2.0 g L^{-1} NaCl or 2.0 g L^{-1} Na_2SO_4	4.5	30	44
TFC-2	0.6 MPa<br>2.0 g L^{-1} NaCl or 2.0 g L^{-1} Na_2SO_4	54.1	210	42
TFC	0.4 MPa<br>1.0 g L^{-1} NaCl or 1.0 g L^{-1} Na_2SO_4	64.6	145	45
TFN-AU1	0.4 MPa<br>1.0 g L^{-1} NaCl or 1.0 g L^{-1} Na_2SO_4	43.6	180	45
TFN-AU2	0.4 MPa<br>1.0 g L^{-1} NaCl or 1.0 g L^{-1} Na_2SO_4	41.3	21.3	45
TFN-AU3	0.4 MPa<br>1.0 g L^{-1} NaCl or 1.0 g L^{-1} Na_2SO_4	33.6	268	45
TFN-AU4	0.4 MPa<br>1.0 g L^{-1} NaCl or 1.0 g L^{-1} Na_2SO_4	31.4	308	45
SWCNT (3 cycles)	0.6 MPa<br>1.0 g L^{-1} NaCl or 1.0 g L^{-1} Na_2SO_4	24.7	442	45
PA/PAN	0.2 MPa<br>1.0 g L^{-1} NaCl or 1.0 g L^{-1} Na_2SO_4	31.6	119.6	46
PA/PDA/PAN	0.2 MPa<br>1.0 g L^{-1} NaCl or 1.0 g L^{-1} Na_2SO_4	30.5	163.9	46
PA/PDA-COF(3)/PAN	0.2 MPa<br>1.0 g L^{-1} NaCl or 1.0 g L^{-1} Na_2SO_4	12.2	207	46
TFC	0.6 MPa<br>1.0 g L^{-1} NaCl or 1.0 g L^{-1} Na_2SO_4	9.2	103	47
TFN-4H	0.6 MPa<br>1.0 g L^{-1} NaCl or 1.0 g L^{-1} Na_2SO_4	10.9	194	47
TFN-4S	0.6 MPa<br>1.0 g L^{-1} NaCl or 1.0 g L^{-1} Na_2SO_4	9	134	47
Freestanding polyamide	0.4 MPa<br>1.5 g L^{-1} NaCl or 1.5 g L^{-1} Na_2SO_4	80.6	251	48
PD/ZIF-8 mass loading of 4.3 µg cm^{-2}	0.4 MPa<br>1.0 g L^{-1} NaCl or 1.0 g L^{-1} Na_2SO_4	18.6	535	49
PEI-0.03 wt%	0.5 MPa<br>1.5 g L^{-1} NaCl or 1.5 g L^{-1} Na_2SO_4	3.3	480	50
PEI-0.05 wt%	0.5 MPa<br>1.5 g L^{-1} NaCl or 1.5 g L^{-1} Na_2SO_4	5.0	240	50
PEI-0.1 wt%	0.5 MPa<br>1.5 g L^{-1} NaCl or 1.5 g L^{-1} Na_2SO_4	3.2	186	50
TS-I	0.48 MPa<br>2.0 g L^{-1} NaCl or 2.0 g L^{-1} Na_2SO_4	54.2	133	51

TS-II	0.48 MPa<br>2.0 g L^{-1} NaCl or 2.0 g L^{-1} Na_2SO_4	126.0	258	[51]
TFC_0	0.3 MPa<br>10 mmol NaCl or<br>10 mmol Na_2SO_4	8.4	68	[52]
TFC_{50}	0.3 MPa<br>10 mmol NaCl or<br>10 mmol Na_2SO_4	35.5	98	[52]
TFC_{90}	0.3 MPa<br>10 mmol NaCl or<br>10 mmol Na_2SO_4	4.4	202	[52]
sMIP PES-PSA5	1 MPa<br>2.0 g L^{-1} NaCl or 2.0 g L^{-1} Na_2SO_4	80.6	37	[53]
PIP/dopamine: 0	0.4 MPa<br>1.0 g L^{-1} NaCl or 1.0 g L^{-1} Na_2SO_4	2.8	175	[54]
PIP/dopamine: 1.0	0.4 MPa<br>1.0 g L^{-1} NaCl or 1.0 g L^{-1} Na_2SO_4	15.2	135	[54]
PIP/dopamine: 2.5	0.4 MPa<br>1.0 g L^{-1} NaCl or 1.0 g L^{-1} Na_2SO_4	23.8	108	[54]
TFCPIP-0	0.6 MPa<br>2.0 g L^{-1} NaCl or 2.0 g L^{-1} Na_2SO_4	34.4	32	[55]
TFCMA-0	0.6 MPa<br>2.0 g L^{-1} NaCl or 2.0 g L^{-1} Na_2SO_4	14.5	53	[55]
TFNMA-GO	0.6 MPa<br>2.0 g L^{-1} NaCl or 2.0 g L^{-1} Na_2SO_4	11.9	90	[55]
PA@EDA 0.15%	0.6 MPa<br>2.0 g L^{-1} NaCl or 2.0 g L^{-1} Na_2SO_4	37.6	42	[12]
PA@EDA 1%	0.6 MPa<br>2.0 g L^{-1} NaCl or 2.0 g L^{-1} Na_2SO_4	8.2	11	[12]
PA@EDA 2%	0.6 MPa<br>2.0 g L^{-1} NaCl or 2.0 g L^{-1} Na_2SO_4	1.9	6	[12]
PA@DCA 0.2%	0.6 MPa<br>2.0 g L^{-1} NaCl or 2.0 g L^{-1} Na_2SO_4	58.3	83	[12]
PA@DCA 1.5%	0.6 MPa<br>2.0 g L^{-1} NaCl or 2.0 g L^{-1} Na_2SO_4	23.6	15	[12]
PA@DCA 2.5%	0.6 MPa<br>2.0 g L^{-1} NaCl or 2.0 g L^{-1} Na_2SO_4	17.4	13	[12]
TMC−PIP	1 MPa<br>2.0 g L^{-1} NaCl or 2.0 g L^{-1} Na_2SO_4	44.5	63	[56]
C-TMC−MPD	1 MPa<br>2.0 g L^{-1} NaCl or 2.0 g L^{-1} Na_2SO_4	3.1	78	[56]
BTC−PIP	1 MPa<br>2.0 g L^{-1} NaCl or 2.0 g L^{-1} Na_2SO_4	18.5	97	[56]

m-XDA 100 (M1)	1 MPa 2.0 g L^{-1} NaCl or 2.0 g L^{-1} Na_2SO_4	14.0	39	[57]
m-XDA 80 (M2)	1 MPa 2.0 g L^{-1} NaCl or 2.0 g L^{-1} Na_2SO_4	22.5	42	[57]
m-XDA 60 (M3)	1 MPa 2.0 g L^{-1} NaCl or 2.0 g L^{-1} Na_2SO_4	20.9	46	[57]
m-XDA 40 (M4)	1 MPa 2.0 g L^{-1} NaCl or 2.0 g L^{-1} Na_2SO_4	24.2	52	[57]
m-XDA 20 (M5)	1 MPa 2.0 g L^{-1} NaCl or 2.0 g L^{-1} Na_2SO_4	19.3	64	[57]
m-XDA 0 (M6)	1 MPa 2.0 g L^{-1} NaCl or 2.0 g L^{-1} Na_2SO_4	17.5	89	[57]
GO/PAN membrane	0.2 MPa 10 mmol NaCl or 10 mmol Na_2SO_4	2.3	162	[58]
$(PEI\text{-}PSS)_2$	0.17 MPa 2 mmol NaCl or 2 mmol Na_2SO_4	1.8	80	[59]
$(PDADMAC\text{-}PSS)_2$	0.17 MPa 2 mmol NaCl or 2 mmol Na_2SO_4	4.2	190	[59]
$(PDADMAC\text{-}PSS)_4$	0.17 MPa 2 mmol NaCl or 2 mmol Na_2SO_4	9.4	140	[59]
$(PDADMAC\text{-}PSS)_6$	0.17 MPa 2 mmol NaCl or 2 mmol Na_2SO_4	11.1	130	[59]
PI-NF after imidization	0.6 MPa 1.0 g L^{-1} NaCl or 1.0 g L^{-1} Na_2SO_4	9.4	40	[60]
PDA/PEI/10 min	0.6 MPa 1.0 g L^{-1} NaCl or 1.0 g L^{-1} Na_2SO_4	2.3	178	[61]
Piperazine (PIP)	0.6 MPa 1.0 g L^{-1} NaCl or 1.0 g L^{-1} Na_2SO_4	34.5	53	[62]
PIP/NH_2-PEG-NH_2 2 kDa	0.6 MPa 1.0 g L^{-1} NaCl or 1.0 g L^{-1} Na_2SO_4	83.4	58	[62]
PIP/NH_2-PEG-NH_2 (After NaClO treatment)	0.6 MPa 2.0 g L^{-1} NaCl or 2.0 g L^{-1} Na_2SO_4	23.6	56	[62]
Piperazine (PIP)	0.6 MPa 1.0 g L^{-1} NaCl or 1.0 g L^{-1} Na_2SO_4	14.9	58	[63]
PIP + ABA	0.6 MPa 1.0 g L^{-1} NaCl or 1.0 g L^{-1} Na_2SO_4	12.4	119	[63]
OH-β-CDs	0.3 MPa	35.5	97	[52]

incorporated TFC	10 mmol L^{-1} Na_2SO_4 or 10 mmol L^{-1} NaCl			
CNC incorporated TFC	0.6 MPa<br>2.0 g L^{-1} NaCl or 2.0 g L^{-1} Na_2SO_4	59.5	165	64
Sericin-TMC TFC	0.6 MPa<br>0.5 g L^{-1} NaCl or 0.5 g L^{-1} Na_2SO_4	12.9	119	665
CNC-TFC-Ms	0.6 MPa<br>2.0 g L^{-1} NaCl or<br>2.0 g L^{-1} Na_2SO_4	60	168 to 2.0 g L^{-1} Na_2SO_4	66

Supplementary Table 4 | Li^+/Mg^{2+} separation selectivity ($\alpha_{Li^+/Mg^{2+}}$) and water flux of the A-PA, A-PA/DA-G4D, A-PA/BA-G4D and A-PA/p-HC-G4D PA membranes. Test condition: 1 MPa, 25°C, 7.5 LPM.

Membrane	Feed solution	Ion rejection rate (%)		$\alpha_{Li^+/Mg^{2+}}$
		Li^+	Mg^{2+}	
A-PA	7.8[a]	18.43	94.85	15.84
	15.6[b]	2.30	94.54	10.44
	31.2[c]	–67.26	90.65	17.89
	31.2@1.5 MPa[d]	–6.43	92.72	14.62
A-PA/DA-G4D	7.8[a]	11.90	98.03	44.72
	15.6[b]	–3.45	98.91	94.91
	31.2[c]	–70.734	97.20	60.98
	31.2@1.5 MPa[d]	–24.88	96.91	40.41
A-PA/BA-G4D	7.8[a]	17.44	99.15	97.13
	15.6[b]	–10.402	98.83	94.16
	31.2[c]	–71.70	98.53	116.8
	31.2@1.5 MPa[d]	–12.43	98.82	95.28
A-PA/p-HC-G4D	7.8[a]	12.56	98.64	64.29
	15.6[b]	–9.34	98.41	68.77
	31.2[c]	–60.98	97.77	72.19
	31.2@1.5 MPa[d]	21.34	97.85	36.59

a Feed solution composed of 0.5 g L^{-1} LiCl and 2.5 g L^{-1} $MgCl_2$, demonstrating containing 81.87 ppm of Li^+, 638.19 ppm of Mg^{2+} and 2285.9 ppm of Cl^-, respectively.

b Feed solution composed of 0.5 g L^{-1} LiCl and 5.0 g L^{-1} $MgCl_2$, demonstrating containing 81.87 ppm of Li^+, 1276.38 ppm of Mg^{2+} and 4153.7 ppm of Cl^-, respectively.

c Feed solution composed of 0.5 g L^{-1} LiCl and 10.0 g L^{-1} $MgCl_2$, demonstrating containing 81.87 ppm of Li^+, 2552.75 ppm of Mg^{2+} and 7889.3 ppm of Cl^-, respectively.

d Feed solution composed of 0.5 g L^{-1} LiCl and 10.0 g L^{-1} $MgCl_2$ was operated at 1.5 MPa, demonstrating containing 81.87 ppm of Li^+, 2552.75 ppm of Mg^{2+} and 7889.3 ppm of Cl^-, respectively.

Supplementary Table 5 | Literature data and the obtained results from this work in terms of Li^+/Mg^{2+} separation.

Membrane Type	Mg^{2+} conc. (ppm)	Li^+ conc. (ppm)	Mg^{2+}/Li^+ mass ratio (MLR)	Mg^{2+} rejection (%)	Li^+ rejection (%)	$\alpha_{Li^+/Mg^{2+}}$	Ref.
Desal DL	9120	152	60	65	–80	5.1	67
Desal DL	5785	132	43.8	60	–10	2.8	67
NF 270	2820	141	20	80	45	2.8	68
NF 90	2820	141	20	94	87	2.2	68
NF 270	705	141	5	89	33	6.1	68
NF 90	705	141	5	99.5	62	76	68
NF 270	1410	141	10	85	41	3.9	68
NF 90	1410	141	10	99	65	35	68
NF 270	2115	141	15	86	45	3.9	68
NF 90	2115	141	15	98	77.5	11.3	68
NFX	469.3	23.5	20	89.1	–84.1	16.9	69
NFX	486.6	12.2	40	73.7	–72.8	6.6	69
NFX	492.7	8.2	60	85.5	–122.2	15.4	69
NFX	938.5	46.9	20	86.2	–79.7	13.1	69
NFX	1407.8	70.4	20	86.3	–89.8	13.9	69
NF 90	469.3	23.5	20	96.6	28.8	20.8	69
NF 90	486.6	12.2	40	94.9	3.2	19.2	69
NF 90	492.7	8.2	60	94.9	5.1	18.5	69
NF 90	938.5	46.9	20	96.1	15.6	21.7	69
NF 90	1407.8	70.4	20	95.8	4.7	22.7	69
NF 270	469.3	23.5	20	51.7	–10.2	2.3	69
NF 270	486.6	12.2	40	38.8	–8	1.8	69
NF 270	492.7	8.2	60	46.9	–12.2	2.1	69
NF 270	938.5	46.9	20	51.9	–10.2	2.3	69
NF 270	1407.8	70.4	20	49.9	–15.4	2.3	69
PEI/GODs-NH2/TMC	469.3	23.5	20	97	20	26.7	70
Dual-skin layer NF (PEI-g-PA (+)	469.3	23.5	20	97.2	6.5	33.4	71
Dual-skin layer NF	438.1	43.8	10	97.3	7	34.4	71
Dual-skin layer NF	486.6	12.2	40	97.3	6.5	34.6	71

Dual-skin layer NF	938.5	46.9	20	96.7	6.7	28.3	71
Dual-skin laver NF	1407.8	70.4	20	96.1	6	24.1	71
(PES-GO)/PEI/TMC	469.3	23.5	20	95.1	22	15.9	72
PES/CNC-COOH/PA	480.7	16	30	96.1	21.8	20.1	73
PES/CNC-COOH/PA	492.7	8.2	60	95.6	11.6	20	73
PEI-TMC	469.3	23.5	20	95	19	16.2	74
PIP-MWCNTs/PEI/TMC	471.5	22	21.4	95	18	16.4	75
PA-B2-E3 PEI	2400	100	24	92.3	29	9.2	76
MWCNTs-COOK (PEI)	469.3	23.5	20	98.6	21.6	57.7	77
PES/CODs-NH2-TMC (PEI)	480.7	16	30	94.7	22.9	14.4	78
SPE-PEI600	500.1	3.3	150	89.1	–30.9	12	79
SPE-PEI600	1250.4	8.3	150	84.7	–38.1	9	79
SPE-PEI600	2500.7	16.7	150	79.6	–43.1	7	79
TFN/PEI-3	469.3	23.5	20	97.4	5	36.5	80
PAA/TMC (+)	1594.4	405.63	20	99.1	23	82.8	81
PEI + DTES/TMC	504.14	16.51	20	91.46	–10.5	12.95	82
QBPD membrane (+)	-	-	50	-	-	5.2	83
PEI@15C5-TMC (+)	-	-	20	-	-	14	84
QEDTP NFM (+) PEI	1974.88	25.12	120	-	-	15.6	85
GLIP-5 (+) Ethylenediamine (EDA)	400	20	20	-	-	28	85
GLIP-6 (+) Ethylenediamine (EDA)	20	20	1	-	-	34.35	85
GLIP-7 (+) Ethylenediamine (EDA)	800	20	40	-	-	20.71	85
DAIB (+) PEI	2552.72	81.87	31.2	-	-	11.1	86
DAIB (+) PEI	2552.72	81.87	31.2	-	-	5.7	86
N-CPTC–TAEA	2552.72	81.87	15.6	-	-	25.94	87
N-CPTC–TAEA	2552.72	81.87	31.2	-	-	36.5	87
PEI-LDH/GA	867.57	132.43	10	-	-	18.7	88
SIP-0.15	468.39	23.42	20			15.38	89
Janus PEI/PIP-TMC	-	-	-	-	-	18.26	90
polyamide-TG-8	468.39	23.42	20			83	91
PSS/PAH2.5 (LbL)	463	27	20	99.9	58.6	279	92
PSS/PAH2.5 (LbL)	468	12.6	40	99.9	57.6	370.3	92
PSS/PAH2.5 (LbL)	477	8.6	60	99.9	58	381.6	92
PSS/PAH2.5 (LbL)	892	47.5	20	99.7	24.6	250.2	92
PSS/PAH2.5 (LbL)	1230	60	20	99.4	–40.8	249.2	92
PSS/PAH2.5 (LbL)	1310	66	20	99	–55	153.2	92
PSS/PAH2.5-X (LbL)	468	23.8	20	99.7	22.1	262.3	92
PSS/PAH2.5-X(LbL)	500	13.8	40	99.7	29.6	255.2	92
PSS/PAH2.5-X (LbL)	506	9	60	99.8	34	330.2	92

PSS/PAH2.5-X (LbL)	957	49.8	20	99.4	–13.8	196.2	[92]
PSS/PAH2.5-X(LbL)	1230	60	20	99.1	–55.8	176.7	[92]
PSS/PAH2.5-X (LbL)	1530	86	20	98.5	–90.7	126	[92]
PES/PSS/PAH (LbL)	469.3	23.5	20	99	28.6	71.4	[93]
PES/PSS/PAH (LbL)	486.6	12.2	40	98.9	28	65.5	[93]
PES/PSS/PAH (LbL)	492.7	8.2	60	99	25.7	74.3	[93]
PES/PSS/PAH (LbL)	938.5	46.9	20	98	–22.1	61	[93]
PES/PSS/PAH (LbL)	1407.8	70.4	20	96.5	–60.2	45.8	[93]
PES/(PIP-PHF)/TMC (inorganic doped PA)	471.5	22	21.4	91.5	–10	12.9	[94]
MCPM-2.0 (inorganic doped TMC-PIP PA)	126	81.98	1.54	93.83	16.8	13.46	[95]
TMC/TMDMA–PIP-15 (organic doped TMC-PIP PA)	2552.72	81.87	31.2	97.42	–51.36	58.67	[96]
TMC/TMDMA–PIP-15 (organic doped TMC-PIP PA)	2552.72	81.87	15.6	96.83	–11.6	35.2	[96]
TMC/TMDMA–PIP-15 (organic doped TMC-PIP PA)	2552.72	81.87	31.2	97.15	–49.83	52.57	[96]
TMC/TMDMA–PIP-15 (organic doped TMC-PIP PA)	2552.72	81.87	31.2	96.7	–43.7	43.54	[96]
HACC/PIP-TMC (organic doped TMC-PIP PA)	470.37	6.59	71.38	-	-	115	[97]
NF-IL-2% (TMC-PIP modified)	252.6	16.5	15.3	86.5	–26	9.3	[98]
NF-IL-2% (TMC-PIP modified)	505.3	16.5	30.7	82	–45	8.1	[98]
NF-IL-2% (TMC-PIP modified)	757.9	16.5	46	80	–49	7.5	[98]
NF-IL-2% (TMC-PIP modified)	1010.5	16.5	61.4	75	–55	6.2	[98]
PAN/DAPP/TMC (Other)	469.3	23.5	20	46	–40.7	2.6	[99]
MBCN-0.02（Other）	494.9	6.8	73	97.5	37.5	25	[100]
MBCN-0.02 (Other)	1484.6	20.3	73	92	–50	18.8	[100]
Cu-MPD (Other)	474.3	20.2	23.5	91.5	32.3	8	[101]
COFMs (Other)	1979.85	20.15	150	-	–	30.2	[102]
RIP-0.250 membrane (Other)	468.39	23.42	20			9.22	[103]

A-PA	638.18	81.87	7.8	94.85	18.43	15.84	This work
A-PA	1276.36	81.87	15.6	94.54	2.3	10.44	This work
A-PA	2552.72	81.87	31.2	90.65	–67.26	17.89	This work
A-PA @ 1.5 MPa	2552.72	81.87	31.2	92.72	–6.43	14.62	This work
A-PA/DA-G4D	638.18	81.87	7.8	98.03	11.9	44.72	This work
A-PA/DA-G4D	1276.36	81.87	15.6	98.91	–3.45	94.91	This work
A-PA/DA-G4D	2552.72	81.87	31.2	97.2	–70.734	60.98	This work
A-PA/DA-G4D @ 1.5 MPa	2552.72	81.87	31.2	96.91	–24.88	40.41	This work
A-PA/BA-G4D	638.18	81.87	7.8	99.15	17.44	97.13	This work
A-PA/BA-G4D	1276.36	81.87	15.6	98.83	–10.402	94.16	This work
A-PA/BA-G4D	2552.72	81.87	31.2	98.53	–71.7	116.8	This work
A-PA/BA-G4D @ 1.5 MPa	2552.72	81.87	31.2	98.82	–12.43	95.28	This work
A-PA/p-HC-G4D	638.18	81.87	7.8	98.64	12.56	64.29	This work
A-PA/p-HC-G4D	1276.36	81.87	15.6	98.41	–9.34	68.77	This work
A-PA/p-HC-G4D	2552.72	81.87	31.2	97.77	–60.98	72.19	This work
A-PA/p-HC-G4D @ 1.5 MPa	2552.72	81.87	31.2	97.85	21.34	36.59	This work

Supplementary Table 6 | Water permeability, fitted Li^+/Mg^{2+} permeabilities*, Li^+/Mg^{2+} permeability ratios and Li^+/water permeability ratios for the literature reported membranes and the SADs polyamide membranes tested in this work.

Membrane Type	P_w (LMH bar^{-1})	Operation pressure (MPa)	P_{Li^+} (μm s^{-1})	$P_{Mg^{2+}}$ (μm s^{-1})	P_{Li^+}/P_w (bar)	$P_{Li^+}/P_{Mg^{2+}}$	Ref.
Desal DL	6.1	2	32.2	1.21	19	26.6	[67]
Desal DL	6.1	2	39.1	5.14	23	7.6	[67]
NF270	19.7	0.8	4.9	1.11	0.9	4.5	[68]

NF90	3.3	0.8	0	0	0	2.8	68
NF270	19.7	0.8	21.3	2.03	3.9	10.5	68
NF90	3.3	0.8	1.4	0.01	1.5	112.2	68
NF270	19.7	0.8	13.8	2.12	2.5	6.5	68
NF90	3.3	0.8	0.8	0.01	0.8	51.5	68
NF270	19.7	0.8	7.5	1.18	1.4	6.3	68
NF90	3.3	0.8	0.2	0.01	0.2	16.2	68
NFX	2.1	0.8	7	0.15	12	46.4	69
NFX	2.1	0.8	10	0.42	17	23.7	69
NFX	2.1	0.8	8.1	0.19	13.9	42.9	69
NFX	2.1	0.8	7.2	0.18	12.3	39.9	69
NFX	2.1	0.8	6.2	0.14	10.6	45.4	69
NF90	3.3	0.8	1.6	0.05	1.8	32.9	69
NF90	3.3	0.8	2.1	0.07	2.3	31.3	69
NF90	3.3	0.8	2	0.07	2.2	29.8	69
NF90	3.3	0.8	1.6	0.04	1.7	35.6	69
NF90	3.3	0.8	1.2	0.03	1.3	38.3	69
NF270	19.7	0.8	65.7	8.86	12	7.4	69
NF270	19.7	0.8	127.5	18.35	23.3	6.9	69
NF270	19.7	0.8	80.9	10.9	14.8	7.4	69
NF270	19.7	0.8	64.3	38.58	11.8	7.5	69
NF270	19.7	0.8	66.4	7.8	12.1	8.5	69
PEI/GODs-NH2/TMC	11.9	0.3	3	0.07	0.9	42.8	70
Dual-skin layer NF (PEI-g-PA (+)	12	0.4	5.6	0.1	1.7	54.1	71
Dual-skin layer NF	12	0.4	6.7	0.11	2	58.9	71
Dual-skin layer NF	12	0.4	5	0.09	1.5	54.5	71
Dual-skin layer NF	12	0.4	3.3	0.07	1	46.4	71
Dual-skin laver NF	12	0.4	0.8	0.02	0.2	40.3	71
(PES-GO)/PEI/TMC	11.2	0.3	2.9	0.11	0.9	25.9	72
PES/CNC-COOH/PA	4.2	0.8	3.9	0.12	3.3	31.7	73
PES/CNC-COOH/PA	3.4	0.8	3.5	0.11	3.7	31.7	73
PEI-TMC	5	0.8	5.3	0.2	3.8	26.6	74
PIP-MWCNTs/PEI/TMC	14	0.4	8.4	0.32	2.2	26.4	75
PA-B2-E3 PEI	1.5	1	0.9	0.06	2.1	15	76
MWCNTs-COOK (PEI)	12.3	0.4	2.8	0.03	0.8	90.9	77
PES/CODs-NH2-TMC (PEI)	33	0.25	3.4	0.15	0.4	23	78

SPE-PEI600	10	0.6	13	0.6	4.7	21.6	[79]
SPE-PEI600	10	0.6	11	0.61	3.9	18	[79]
SPE-PEI600	10	0.6	3.3	0.21	1.2	16.1	[79]
TFN/PEI-3	30.6	0.4	13.5	0.23	1.6	60.5	[80]
PEI+DTES/TMC	4.96	0.8	11.70	0.3072	6.80	38.09	[82]
PSS/PAH2.5 (LbL)	7.9	0.4	0.9	0.0021	0.4	420.2	[92]
PSS/PAH2.5 (LbL)	7.9	0.4	0.9	0.0015	0.4	568.3	[92]
PSS/PAH2.5 (LbL)	7.9	0.4	0.8	0.0014	0.4	584.4	[92]
PSS/PAH2.5 (LbL)	7.9	0.4	1.6	0.0041	0.7	393.3	[92]
PSS/PAH2.5 (LbL)	7.9	0.4	2.1	0.0047	1	445.2	[92]
PSS/PAH2.5 (LbL)	7.9	0.4	2.7	0.0093	1.2	287.7	[92]
PSS/PAH2.5-X (LbL)	6.2	0.4	1.4	0.0035	0.8	406.8	[92]
PSS/PAH2.5-X (LbL)	6.2	0.4	1.1	0.0029	0.6	383	[92]
PSS/PAH2.5-X (LbL)	6.2	0.4	1	0.002	0.6	495	[92]
PSS/PAH2.5-X (LbL)	6.2	0.4	2.1	0.0063	1.2	325.8	[92]
PSS/PAH2.5-X (LbL)	6.2	0.4	2.1	0.0063	1.2	330.5	[92]
PSS/PAH2.5-X (LbL)	6.2	0.4	2.4	0.0082	1.4	289.8	[92]
PES/PSS/PAH (LbL)	18.4	0.4	4.5	0.0413	0.9	110	[93]
PES/PSS/PAH(LbL)	18.4	0.4	4.1	0.0411	0.8	99.8	[93]
PES/PSS/PAH(LbL)	18.4	0.4	4.1	0.0362	0.8	112.6	[93]
PES/PSS/PAH(LbL)	18.4	0.4	5.2	0.0504	1	103.8	[93]
PES/PSS/PAH(LbL)	18.4	0.4	6.5	0.0729	1.3	88.5	[93]
PES/(PIP-PHF)/TMC (inorganic doped PA)	6.7	0.6	9.1	0.39	4.9	23.2	[94]
MCPM-2.0 (inorganic doped TMC-PIP PA)	15.5	0.2	0.16	1.33	35.53	0.16	[95]
TMC/TMDMA–PIP-15 (organic doped TMC-PIP PA)	17.47	1	19.86	0.19	4.09	105.71	[96]
TMC/TMDMA–PIP-15 (organic doped TMC-PIP PA)	23.28	1	17.14	0.29	2.65	58.74	[96]
TMC/TMDMA–PIP-15 (organic	14.783	1	16.78	0.18	4.09	94.89	[96]

doped TMC-PIP PA)							
TMC/TMDMA–PIP-15 (organic doped TMC-PIP PA)	13.692	1	14.87	0.19	3.91	78.11	96
TMC/TMDMA–PIP-15 (organic doped TMC-PIP PA)	23.271	1	35.18	0.52	5.44	67.98	96
TMC/TMDMA–PIP-15 (organic doped TMC-PIP PA)	22.409	1	33.88	0.50	5.44	67.98	96
NF-IL-2% (TMC-PIP modified)	5.5	0.6	12.3	0.61	8	20	98
NF-IL-2% (TMC-PIP modified)	5.5	0.6	13	0.68	8.5	19	98
NF-IL-2% (TMC-PIP modified)	5.5	0.6	12.4	0.68	8.1	18.2	98
NF-IL-2% (TMC-PIP modified)	5.5	0.6	14.4	0.83	9.4	17.4	98
PAN/DAPP/TMC (Other)	2.6	0.3	21.8	0.85	30.6	26.7	99
MBCN-0.02 （Other）	5.6	0.4	1.5	0.04	0.9	38	100
MBCN-0.02 (Other)	5.6	0.4	0.8	0.02	0.5	34.4	100
Cu-MPD (Other)	16.2	0.5	8.4	0.66	1.9	12.8	101
A-PA	37.99	1	5.07	0.046	0.48	109.28	This work
A-PA	37.99	1	26.10	0.046	2.47	566.17	This work
A-PA	37.99	1	74.78*	1.88*	7.09	39.73	This work
A-PA/DA-G4D	26.43	1	13.56	0.038	1.85	359.75	This work
A-PA/DA-G4D	26.43	1	71.54	0.020	9.74	3668.51	This work
A-PA/DA-G4D	26.43	1	37.46*	0.32*	5.10	116.19	This work
A-PA/BA-G4D	23.29	1	11.12	0.020	1.72	561.72	This work
A-PA/BA-G4D	23.29	1	100.23	0.00023	15.50	444273.94	This work
A-PA/BA-G4D	23.29	1	30.92*	0.14*	4.78	216.21	This work

A-PA/p-HC-G4D	26.75	1	12.87	0.025	1.73	508.51	This work
A-PA/p-HC-G4D	26.75	1	41.22*	0.34*	5.55	120.17	This work
A-PA/p-HC-G4D	26.75	1	33.11*	0.25*	4.46	132.25	This work

Note: * indicate that, in order to fit out the P_{Li^+} (μm s^{-1}) and $P_{Mg^{2+}}$ (μm s^{-1}), we simplify the SDEM model by ignoring the effect of concentration polarization on the permeance.

Supplementary Table 7 | The representative reported data for each type of membrane are chosen to conduct the module analysis, then we calculated Li recovery vs. water recovery along a simulated module. The following are the specific data used. Note: indicate that, in order to fit out the P_{Li^+} (μm s^{-1}) and $P_{Mg^{2+}}$ (μm s^{-1}), we simplify the SDEM model by ignoring the effect of concentration polarization on the permeance.

Membrane Type	Pw (L m^{-2} h^{-1} bar^{-1})	P_{Li^+} (μm s^{-1})	$P_{Mg^{2+}}$ (μm s^{-1})	Ref.
A-PA/DA-G4D	26.43	46.46	0.59	This work
A-PA/BA-G4D	23.29	36.63	0.22	This work
A-PA/p-HC-G4D	26.75	46.67	0.41	This work
NF90	3.3	1.4	0.01	[68]
PAN/DAPP/TMC	2.6	21.8	0.85	[99]
Desal DL	6.1	39.1	5.14	[67]
Dual-skin layer NF	12.0	6.7	0.11	[71]
PES/PSS/PAH (LbL)	18.4	6.5	0.0729	[93]
MBCN-0.02	5.6	1.5	0.04	[100]
PES/CNC-COOH/PA	4.2	3.9	0.12	[73]
SPE-PEI600	10.0	11.0	0.61	[79]
TFN/PEI-3	30.6	13.5	0.23	[80]
Cu-MPD	16.2	8.4	0.66	[101]
PA-B2-E3	1.5	0.9	0.06	[76]
PSS/PAH2.5 (LbL)	2.7	0.0093	7.9	[92]

Supplementary Table 8 | Materialized parameters of the common cations and anions[104–106].

Ion	Ionic radius (nm)	Hydration Number (±1)	Hydrated radius (nm)	Diffusion coefficient (10^{-9} m^2/s)	Hydration free energy (kJ mol^{-1})	Lifetime/exchange rate (s)
Mg^{2+}	0.065	6	0.428	0.706	1828	10^{-6}
Cl^{-}	0.181	1	0.332	2.032	340	$\sim10^{-11}$
SO_4^{2-}	0.290	-	0.379	1.065	1145	-
Li^{+}	0.068	5	0.382	1.03	474	5×10^{-9}

4 Supplementary Notes

Matlab Codes for the calculation of P_{Li^+} and $P_{Mg^{2+}}$[69,107]

```
% (1) Main
function [pLilist, pMglist] = main (cfLi, cfMg, jv, RLi, RMg)
% units: mM, mM, LMH, 0-1, 0-1
CfLi = [11.7 11.7 11.7];
cfMg = [26.59 106.36 26.59];
RLi = [0.1843 0.2134 0.1256];
RMg = [0.9485 0.9785 0.9864];
Jv = [379.91,267.54,232,86]/3.6; %μm/s
k=100; %LMH
pLilist = zeros (1, length(cfLi));
pMglist = zeros (1, length(cfMg));
for i = 1:3
    x0 = [100, 5]*1e4; % initial guess
    options = optimset ('Display','iter');
    f = @(x)mainfitting5(x, cfLi(i), cfMg(i), jv(i), RLi(i), RMg(i), k);
    p = lsqnonlin(f,x0,zeros(size(x0)),[],options); % solve nonlinear regression
    pLilist(i) = p(1)/1e4;
    pMglist(i) = p(2)/1e4;
end
end

% (2) Fitting Li/Mg rejections with SDEM model
function y = mainfitting5(x, cfLi, cfMg, jv, RLi, RMg, k)
% units: mM, mM, μm/s, 0-1, 0-1, LMH
c1 = cfLi*(1-RLi+RLi*exp(jv*3.6/k)); % Li conc at feed surface
c2 = cfMg*(1-RMg+RMg*exp(jv*3.6/k)); % Mg conc at feed surface
```

```
xf = [jv, c1, c2];
R = SDEM (x/1e4, xf);
r1 = 1/((1/RLi-1)*exp(-jv*3.6/k)+1); % Li intrinsic rejection
r2 = 1/((1/RMg-1)*exp(-jv*3.6/k)+1); % Mg intrinsic rejection
y = [1*(R(1)-r1),50*(R(2)-r2)];
end

% (3) SDEM model
function R = SDEM(P, X)
Jv = X(1);
c1 = X(2);
c2 = X(3);
c3 = c1 + 2*c2;

p1 = P(1);
p2 = P(2);
p3 = p1;

z1 = 1;
z2 = 2;
z3 = -1;

c0 = c1+c2+c3;
u0 = (z1^2*c1+z2^2*c2+z3^2*c3)/c0;

pi = z1*z2*z3;
sigma = z1+z2+z3;

b02 = (z2^2-z3^2)/p1-(z1^2-z3^2)/p2+(z1^2-z2^2)/p3;
b12 = z1*(z2^2-z3^2)/p1-z2*(z1^2-z3^2)/p2+z3*(z1^2-z2^2)/p3;
b01 = (z2-z3)/p1-(z1-z3)/p2+(z1-z2)/p3;
b11 = z1*(z2-z3)/p1-z2*(z1-z3)/p2+z3*(z1-z2)/p3;
b_11 = 1/z1*(z2-z3)/p1-1/z2*(z1-z3)/p2+1/z3*(z1-z2)/p3;

fun = @(x)upsolver(x,pi,b02,b12,b01,b11,b_11,u0,c0,z1,z2,z3,Jv);
x0 = -pi*(u0^2*b_11+u0*b02+pi*b01)/(u0^2*b01+u0*b12+pi*b11)+1e-6;
up = fsolve(fun, x0);

m1 = (pi*b02+b12*up)^2-4*pi*(pi*b01+b11*up)*(pi*b_11+b01*up);
m2 = pi*b02+b12*up;
m3 = 2*(pi*b_11+b01*up);
fp = (m1^0.5-m2)/m3;
fm = (m1^0.5+m2)/m3;
```

```
n1 = (fm+u0)/(fm+up);
n2 = fm/(fm+fp);
n3 = (fp-u0)/(fp-up);
n4 = fp/(fm+fp);
cp = c0*n1^n2*n3^n4;

cp1 = cp*(up+z2*z3)/(z1-z2)/(z1-z3);
cp2 = cp*(up+z1*z3)/(z2-z1)/(z2-z3);
cp3 = cp*(up+z2*z1)/(z3-z2)/(z3-z1);

R1 = 1-cp1/c1;
R2 = 1-cp2/c2;
R3 = 1-cp3/c3;
R = [R1, R2];
end

% (4) up solver
function y=upsolver(x, pi, b02,b12,b01,b11,b_11,u0,c0,z1,z2,z3,jv)
up=x;
m1 = (pi*b02+b12*up)^2-4*pi*(pi*b01+b11*up)*(pi*b_11+b01*up);
m2 = pi*b02+b12*up;
m3 = 2*(pi*b_11+b01*up);
fp = (m1^0.5-m2)/m3;
fm = (m1^0.5+m2)/m3;
n1 = (fm+u0)/(fm+up);
n2 = fm/(fm+fp);
n3 = (fp-u0)/(fp-up);
n4 = fp/(fm+fp);
cp = c0*n1^n2*n3^n4;
y=(c0/cp-1)*(z1-z2)*(z2-z3)*(z1-z3)/(pi*b_11+b01*up)-jv;
end
```

Matlab Codes for Module Analysis[69,107]

%(1)Main function

```
function R = module(A)
Pressure = 4; % bar, constant pressure
cf1 = 3.4; % mM, Li feed
cf2 = 19.6; % mM, Mg feed
cf3 = cf1 + 2*cf2; % mM, Cl feed
A = A; % LMH/bar, water permeance

dWR = 0.01; % water recovery step size
WR = 0.7; % target final water recovery
n = WR/dWR; % number of steps
```

```
m = 1; % step indicator

jvlist = zeros(1, n);
cflist1 = zeros(1, n+1);
cflist2 = zeros(1, n+1);
cflist3 = zeros(1, n+1);
cplist1 = zeros(1, n);
cplist2 = zeros(1, n);
cplist3 = zeros(1, n);
cpmlist1 = zeros(1, n);
cpmlist2 = zeros(1, n);
plist1 = zeros(1, n);
plist2 = zeros(1, n);
rlilist = zeros(1, n+1);
%Plist = zeros(1, n+1);
cflist1(m) = cf1;
cflist2(m) = cf2;
cflist3(m) = cf3;

while m<=n
    if m==1
        jvguess = 0.1;
    else
        jvguess = jvlist(m-1)+0.1;
    end

    y = SDEM2([Pressure, cflist1(m), cflist2(m), cflist3(m),jvguess], A);
    cplist1(m) = y(1);
    cplist2(m) = y(2);
    cplist3(m) = y(3);
    cpmlist1(m) = mean(cplist1(1:m));
    cpmlist2(m) = mean(cplist2(1:m));
    cpmlist3(m) = mean(cplist3(1:m));
    rlilist(m+1) = m*dWR*mean(cplist1(1:m))/cf1;
    cflist1(m+1) = ((1-(m-1)*dWR)*cflist1(m)-dWR*cplist1(m))/(1-(m-1)*dWR-dWR);
    cflist2(m+1) = ((1-(m-1)*dWR)*cflist2(m)-dWR*cplist2(m))/(1-(m-1)*dWR-dWR);
    cflist3(m+1) = ((1-(m-1)*dWR)*cflist3(m)-dWR*cplist3(m))/(1-(m-1)*dWR-dWR);

    jvlist(m) = y(4);
    plist1(m) = y(5);
    plist2(m) = y(6);
    m = m+1;
    display(m);
end
```

```
%Rlist1 = 1-cplist1./cflist1(1:n); % Local Li rejection
%Rlist2 = 1-cplist2./cflist2(1:n); % Local Mg rejection
%Slist = (1-Rlist1)./(1-Rlist2); % Local selectivity
%Rover1 = 1-cpmlist1/cf1; % Cumulative Li rejection
%Rover2 = 1-cpmlist2/cf2; % Cumulative Mg rejection
%Sover = (1-Rover1)./(1-Rover2); % Cumulative selectivity
%jvlist = jvlist*3.6; % Flux converted to LMH
%MLR = cflist2./cflist1*(24/7); % MLR

R = rLilist*100;

%(2) SDEM2
function y = SDEM2(X,A)
Pressure = X(1);
c1 = X(2);
c2 = X(3);
c3 = X(4);

z1 = 1; % Li
z2 = 2; % Mg
z3 = -1; % Cl

c0 = c1+c2+c3;
u0 = (z1^2*c1+z2^2*c2+z3^2*c3)/c0;

pi = z1*z2*z3;
sigma = z1+z2+z3;

if X(5) == 0.1
    Jv = A*(Pressure-8.314*298/1e5*(c1*2*0.97+c2*3*0.9643*c2^(-0.025)))/3.6; % μm/s
else
    Jv = X(5);
end
Jv_old = 0;
while abs(Jv-Jv_old)>0.001
    P = [46.6777 0.4084]; % constant permeability;
    p1 = P(1);
    p2 = P(2);
    p3 = p1;

    b02 = (z2^2-z3^2)/p1-(z1^2-z3^2)/p2+(z1^2-z2^2)/p3;
    b12 = z1*(z2^2-z3^2)/p1-z2*(z1^2-z3^2)/p2+z3*(z1^2-z2^2)/p3;

    b01 = (z2-z3)/p1-(z1-z3)/p2+(z1-z2)/p3;
```

```
        b11 = z1*(z2-z3)/p1-z2*(z1-z3)/p2+z3*(z1-z2)/p3;
        b_11 = 1/z1*(z2-z3)/p1-1/z2*(z1-z3)/p2+1/z3*(z1-z2)/p3;

        fun = @(x)upsolver(x,pi,b02,b12,b01,b11,b_11,u0,c0,z1,z2,z3,Jv);
        x0 = -pi*(u0^2*b_11+u0*b02+pi*b01)/(u0^2*b01+u0*b12+pi*b11)+1e-6;
        options = optimset('Display','off');
        up = fsolve(fun,x0,options);

        m1 = (pi*b02+b12*up)^2-4*pi*(pi*b01+b11*up)*(pi*b_11+b01*up);
        m2 = pi*b02+b12*up;
        m3 = 2*(pi*b_11+b01*up);
        fp = (m1^0.5-m2)/m3;
        fm = (m1^0.5+m2)/m3;

        n1 = (fm+u0)/(fm+up);
        n2 = fm/(fm+fp);
        n3 = (fp-u0)/(fp-up);
        n4 = fp/(fm+fp);
        cp = c0*n1^n2*n3^n4;

        cp1 = cp*(up+z2*z3)/(z1-z2)/(z1-z3);
        cp2 = cp*(up+z1*z3)/(z2-z1)/(z2-z3);
        cp3 = cp*(up+z2*z1)/(z3-z2)/(z3-z1);

        % Update for the next iteration
        Jv_old = Jv;
        %c1 = cp1+(X(2)-cp1)*exp(Jv*3.6/100); %concentration polarization neglected
        %c2 = cp2+(X(3)-cp2)*exp(Jv*3.6/100);
        %c3 = c1 + 2*c2;
        %c0 = c1+c2+c3;
        u0 = (z1^2*c1+z2^2*c2+z3^2*c3)/c0;
        Jv_new = A*(Pressure-8.314*298/1e5*(c1*2*0.97+c2*3*0.9643*c2^(-0.025)...
            -cp1*2*0.97-cp2*3*0.9643*c2^(-0.025)))/3.6;%μm/s
        Jv = 0.5*Jv_old+0.5*Jv_new;
    end
    y = [cp1, cp2, cp3, Jv, p1, p2];
    end
```

%(3)upsolver

```
function y = upsolver(x,pi,b02,b12,b01,b11,b_11,u0,c0,z1,z2,z3,Jv)
up = x;
m1 = (pi*b02+b12*up)^2-4*pi*(pi*b01+b11*up)*(pi*b_11+b01*up);
m2 = pi*b02+b12*up;
m3 = 2*(pi*b_11+b01*up);
```

```
fp = (m1^0.5-m2)/m3;
fm = (m1^0.5+m2)/m3;

n1 = (fm+u0)/(fm+up);
n2 = fm/(fm+fp);
n3 = (fp-u0)/(fp-up);
n4 = fp/(fm+fp);
cp = c0*n1^n2*n3^n4;
jn = (c0/cp-1)*(z1-z2)*(z2-z3)*(z1-z3)/(pi*b_11+b01*up);
y = jn-Jv;
end
```

5. Supplementary References

1. Yuan, B., Zhao, S., Hu, P., Cui, J. & Niu, Q. J. Asymmetric polyamide nanofilms with highly ordered nanovoids for water purification. *Nat. Commun.* **11**, 6102 (2020).
2. Washio, I., Shibasaki, Y. & Ueda, M. Facile Synthesis of amine-terminated aromatic polyamide dendrimers via a divergent method. *Org. Lett.* **9**, 1363–1366 (2007).
3. Wang, K. Y. & Chung, T-S. Fabrication of polybenzimidazole (PBI) nanofiltration hollow fiber membranes for removal of chromate. *J. Memb. Sci.* **281**, 307–315 (2006).
4. Wadekar, S. S. & Vidic, R. D. Insights into the rejection of barium and strontium by nanofiltration membrane from experimental and modeling analysis. *J. Membr. Sci.* **564**, 742–752 (2018).
5. Kong, X. et al. High permselectivity hyperbranched polyester/polyamide ultrathin films with nanoscale heterogeneity. *J. Mater. Chem. A* **5**, (2017).
6. Yuan, B. et al. Alicyclic polyamide nanofilms with an asymmetric structure for Cl^{-}/SO_4^{2-} separation. *AIChE J.* **68**, e17419 (2022).
7. Zhang, L. et al. Polyamide nanofiltration membrane with high mono/divalent salt selectivity via pre-diffusion interfacial polymerization. *J. Memb. Sci.* **636**, 119478 (2021).
8. Liang, Y. et al. Polyamide nanofiltration membrane with highly uniform sub-nanometre pores for sub-1 Å precision separation. *Nat. Commun.* **11**, 2015 (2020).
9. Gong, G., Wang, P., Zhou, Z. & Hu, Y. New insights into the role of an interlayer

for the fabrication of highly selective and permeable thin-Film composite nanofiltration membrane. *ACS Appl. Mater. Inter.* **11**, 7349–7356 (2019).

10. Liu, T-Y. et al. Ion-responsive channels of zwitterion-carbon nanotube membrane for rapid water permeation and ultrahigh mono-/multivalent ion selectivity. *ACS nano* **9**, 7488–7496 (2015).
11. Lai, G. S. et al. A practical approach to synthesize polyamide thin film nanocomposite (TFN) membranes with improved separation properties for water/wastewater treatment. *J J. Mater. Chem. A* **4**, 4134–4144 (2016).
12. Ren, D., Bi, X-T., Liu, T-Y. & Wang, X. Oligo-ethylene-glycol based thin-film composite nanofiltration membranes for effective separation of mono-/di-valent anions. *J. Mater. Chem. A* **7**, 1849–1860 (2019).
13. Pan, Y. et al. Enhanced both perm-selectivity and fouling resistance of poly(piperazine-amide) nanofiltration membrane by incorporating sericin as a co-reactant of aqueous phase. *J. Memb. Sci.* **523**, 282–290 (2017).
14. Du, Y., Lv, Y., Qiu, W-Z., Wu, J. & Xu, Z-K. Nanofiltration membranes with narrowed pore size distribution via pore wall modification. *Chem. Commun.* **52**, 8589–8592 (2016).
15. Cao, X-L. et al. The encouraging improvement of polyamide nanofiltration membrane by cucurbituril-based host–guest chemistry. *AIChE J.* **66**, e16879 (2020).
16. Peng, H., Tang, Q., Tang, S., Gong J. & Zhao, Q. Surface modified polyamide nanofiltration membranes with high permeability and stability. *J. Memb. Sci.* **592**, 117386 (2019).
17. Du, Y. et al. Ultrathin alginate coatings as selective layers for nanofiltration membranes with high performance. *ChemSusChem* **10**, 2788–2795 (2017).
18. Qiu, Z-L., Yu, W-H., Shen, Y-J., Zhu, B-K. & Fang, L-F. Janus charged polyamide nanofilm with ultra-high separation selectivity for mono-/divalent ions. *Chem. Eng. J.* **416**, 129023 (2021).
19. Xu, S. et al. Anionic covalent organic framework as an interlayer to fabricate negatively charged polyamide composite nanofiltration membrane featuring ions

sieving. *Chem. Eng. J.* **427**, 132009 (2022).

20. Yuan, B. et al. Polyamide nanofiltration membrane fine-tuned via mixed matrix ultrafiltration support to maximize the sieving selectivity of Li^+/Mg^{2+} and Cl^-/SO_4^{2-}. *Desalination* **538**, 115929 (2022).
21. Han, S. et al. Microporous organic nanotube assisted design of high performance nanofiltration membranes. *Nat. Commun.* **13**, 7954 (2022).
22. Sun, H. et al. Fabrication of thin-film composite polyamide nanofiltration membrane based on polyphenol intermediate layer with enhanced desalination performance. *Desalination* **488**, 114525 (2020).
23. Hao, Y. et al. An ultrahighly permeable-selective nanofiltration membrane mediated by an in situ formed interlayer. *J. Mater. Chem. A* **8**, 5275–5283 (2020).
24. Peng, H. et al. Phosphonium modification leads to ultrapermeable antibacterial polyamide composite membranes with unreduced thickness. *Adv. Mater.* **32**, 2001383 (2020).
25. Shen, K., Li, P., Zhang, T. & Wang, X. Salt-tuned fabrication of novel polyamide composite nanofiltration membranes with three-dimensional turing structures for effective desalination. *J. Memb. Sci.* **607**, 118153 (2020).
26. Zhan, Z-M. et al. Superior nanofiltration membranes with gradient cross-linked selective layer fabricated via controlled hydrolysis. *J. Memb. Sci.* **604**, 118067 (2020).
27. Zhang, Z., Shi, X., Wang, R., Xiao, A. & Wang, Y. Ultra-permeable polyamide membranes harvested by covalent organic framework nanofiber scaffolds: a two-in-one strategy. *Chem. Sci.* **10**, 9077–9083 (2019).
28. Huang, B-Q., Tang, Y-J., Zeng, Z-X. & Xu, Z-L. Microwave heating assistant preparation of high permselectivity polypiperazine-amide nanofiltration membrane during the interfacial polymerization process with low monomer concentration. *J. Memb. Sci.* **596**, 117718 (2020).
29. Ang, MBMY. et al. Improved performance of thin-film nanofiltration membranes fabricated with the intervention of surfactants having different structures for water

treatment. *Desalination* **481**, 114352 (2020).

30. Ji, C., Xue, S., Tang, Y-J., Ma, X-H. & Xu, Z-L. Polyamide membranes with net-like nanostructures induced by different charged MOFs for elevated nanofiltration. *ACS Appl. Polym. Mater.* **2**, 585–593 (2020).
31. Liu, Y. et al. A facile and scalable fabrication procedure for thin-film composite membranes: Integration of phase inversion and interfacial polymerization. *Environ. Sci. Technol.* **54**, 1946–1954 (2020).
32. Yuan, S. et al. Hydrogel assisted interfacial polymerization for advanced nanofiltration membranes. *J. Mater. Chem. A* **8**, 3238–3245 (2020).
33. Yuan, J. et al. Covalent organic framework-modulated interfacial polymerization for ultrathin desalination membranes. *J. Mater. Chem. A* **7**, 25641–25649 (2019).
34. Zeng, Y., Wang, L., Zhang, L. & Yu, J. Q. An acid resistant nanofiltration membrane prepared from a precursor of poly(s-triazine-amine) by interfacial polymerization. *J. Memb. Sci.* **546**, 225–233 (2018).
35. Sun, H. & Wu, P. Tuning the functional groups of carbon quantum dots in thin film nanocomposite membranes for nanofiltration. *J. Memb. Sci.* **564**, 394–403 (2018).
36. Wang, T. et al. Fabrication of high flux nanofiltration membrane via hydrogen bonding based co-deposition of polydopamine with poly(vinyl alcohol). *J. Memb. Sci.* **552**, 222–233 (2018).
37. Li, Y., Wong, E., Mai, Z. & Van der Bruggen, B. Fabrication of composite polyamide/Kevlar aramid nanofiber nanofiltration membranes with high permselectivity in water desalination. *J. Memb. Sci.* **592**, 117396 (2019).
38. Yang. Z. et al. Tannic acid/Fe^{3+} nanoscaffold for interfacial polymerization: Toward enhanced nanofiltration performance. *Environ. Sci. Technol.* **52**, 9341–9349 (2018).
39. Sun, Z. et al. Nanovoid membranes embedded with hollow zwitterionic nanocapsules for a superior desalination performance. *Nano lett.* **19**, 2953–2959 (2019).
40. Wang, Q. et al. Designing high-performance nanofiltration membranes for high-salinity separation of sulfate and chloride in the chlor-alkali process. *Ind. Eng. Chem.*

Res. **58**, 12280–12290 (2019).

41. Jiang. C. et al. Thin-film composite membranes with aqueous template-induced surface nanostructures for enhanced nanofiltration. *J. Memb. Sci.* **589**, 117244 (2019).
42. Jia, L. et al. Polyvinyl alcohol-assisted high-flux thin film nanocomposite membranes incorporated with halloysite nanotubes for nanofiltration. *Environ. Sci-Wat. Res.* **5**, 1412–1422 (2019).
43. Shen, K., Cheng, C., Zhang, T. & Wang, X. High performance polyamide composite nanofiltration membranes via reverse interfacial polymerization with the synergistic interaction of gelatin interlayer and trimesoyl chloride. *J. Memb. Sci.* **588**, 117192 (2019).
44. Rezania, H., Vatanpour, V., Shockravi, A. & Ehsani, M. Study of synergetic effect and comparison of novel sulfonated and carboxylated bulky diamine-diol and piperazine in preparation of negative charge NF membrane. *Sep. Purif. Technol.* **222**, 284–296 (2019).
45. Zhu, J. et al. MOF-positioned polyamide membranes with a fishnet-like structure for elevated nanofiltration performance. *J. Mater. Chem. A* **7**, 16313–16322 (2019).
46. Wu, M. et al. Ultrathin nanofiltration membrane with polydopamine-covalent organic framework interlayer for enhanced permeability and structural stability. *J. Memb. Sci.* **576**, 131–141 (2019).
47. Liao, Z. et al. Hydrophilic hollow nanocube-functionalized thin film nanocomposite membrane with enhanced nanofiltration performance. *ACS Appl Mater Interfaces* **11**, 5344–5352 (2019).
48. Zhu, J. et al. Rapid water transport through controllable, ultrathin polyamide nanofilms for high-performance nanofiltration. *J. Mater. Chem. A* **6**, 15701–15709 (2018).
49. Wang, Z. et al. Nanoparticle-templated nanofiltration membranes for ultrahigh performance desalination. *Nat. Commun.* **9**, 2004 (2018).
50. Trivedi, J. S., Bhalani, D. V., Bhadu, G. R. & Jewrajka, S. K. Multifunctional amines

enable the formation of polyamide nanofilm composite ultrafiltration and nanofiltration membranes with modulated charge and performance. *J. Mater. Chem. A* **6**, 20242–20253 (2018).

51. Tan, Z., Chen, S., Peng, X., Zhang, L. & Gao, C. Polyamide membranes with nanoscale Turing structures for water purification. *Science* **360**, 518–521 (2018).
52. Yao, Z., Guo, H., Yang, Z., Qing, W. & Tang, C. Y. Preparation of nanocavity-contained thin film composite nanofiltration membranes with enhanced permeability and divalent to monovalent ion selectivity. *Desalination* **445**, 115–122 (2018).
53. He, M. et al. High-performance acid-stable polysulfonamide thin-film composite membrane prepared via spinning-assist multilayer interfacial polymerization. *J. Mater. Sci.* **54**, 886–900 (2019).
54. Zhu. J. et al. High-flux thin film composite membranes for nanofiltration mediated by a rapid co-deposition of polydopamine/piperazine. *J. Memb. Sci.* **554**, 97–108 (2018).
55. Xue, S-M., Ji, C-H., Xu, Z-L., Tang, Y-J. & Li, R-H. Chlorine resistant TFN nanofiltration membrane incorporated with octadecylamine-grafted GO and fluorine-containing monomer. *J. Memb. Sci.* **545**, 185–195 (2018).
56. Yuan. B. et al. Ultrathin polyamide membrane with decreased porosity designed for outstanding water-softening performance and superior antifouling properties. *ACS Appl. Mater. Inter.* **10**, 43057–43067 (2018).
57. Liu, Y. et al. Preparation and characterization of a novel nanofiltration membrane with chlorine-tolerant property and good separation performance. *RSC Adv.* **8**, 36430–36440 (2018).
58. Zhang, M., Sun, J., Mao, Y., Liu, G. & Jin, W. Effect of substrate on formation and nanofiltration performance of graphene oxide membranes. *J. Memb. Sci.* **574**, 196–204 (2019).
59. Dizge, N., Epsztein, R., Cheng ,W., Porter, C. J. & Elimelech, M. Biocatalytic and salt selective multilayer polyelectrolyte nanofiltration membrane. *J. Memb. Sci.* **549**,

357–365 (2018).

60. Wei, C. et al. Negatively charged polyimide nanofiltration membranes with high selectivity and performance stability by optimization of synergistic imidization. *J. Memb. Sci.* **563**, 752–761 (2018).

61. Yang, H-C., Wu, M-B., Hou, J., Darling, S. B. & Xu, Z-K. Nanofilms directly formed on macro-porous substrates for molecular and ionic sieving. *J. Mater. Chem. A* **6**, 2908–2913 (2018).

62. Tang, Y-J., Xu, Z-L., Xue, S-M., Wei, Y-M. & Yang, H. Improving the chlorine-tolerant ability of polypiperazine-amide nanofiltration membrane by adding NH_2-PEG-NH_2 in the aqueous phase. *J. Memb. Sci.* **538**, 9–17 (2017).

63. Ang, MBMY. et al. Incorporation of carboxylic monoamines into thin-film composite polyamide membranes to enhance nanofiltration performance. *J. Memb. Sci.* **539**, 52–64 (2017).

64. Bai, L. et al. Incorporation of cellulose nanocrystals (CNCs) into the polyamide layer of thin-Film composite (TFC) nanofiltration membranes for enhanced separation performance and antifouling properties. *Environ. Sci. Technol.* **52**, 11178–11187 (2018).

65. Zhou, C. et al. Thin-film composite membranes formed by interfacial polymerization with natural material sericin and trimesoyl chloride for nanofiltration. *J. Memb. Sci.* **471**, 381–391 (2014).

66. Bai, L. et al. Fabrication and characterization of thin-film composite (TFC) nanofiltration membranes incorporated with cellulose nanocrystals (CNCs) for enhanced desalination performance and dye removal. *Chem. Eng. J.* **358**, 1519–1528 (2019).

67. Sun, S-Y., Cai, L-J., Nie, X-Y., Song, X. & Yu, J-G. Separation of magnesium and lithium from brine using a Desal nanofiltration membrane. *J. Water Process Eng.* **7**, 210–217 (2015).

68. Pramanik, B. K., Asif, M. B., Kentish, S., Nghiem, L. D. & Hai, F. I. Lithium enrichment from a simulated salt lake brine using an integrated nanofiltration-

membrane distillation process. *J. Environ. Chem. Eng.* **7**, 103395 (2019).

69. Wang, R., He, R., He, T., Elimelech, M. & Lin, S. Performance metrics for nanofiltration-based selective separation for resource extraction and recovery. *Nat. Water* **1**, 291–300 (2023).
70. Xu, P., Hong, J., Xu, Z., Xia, H. & Ni, Q-Q. Novel aminated graphene quantum dots (GQDs-NH_2)-engineered nanofiltration membrane with high Mg^{2+}/Li^+ separation efficiency. *Sep. Purif. Technol.* **258**, 118042 (2021).
71. Yang, Z. et al. Dual-skin layer nanofiltration membranes for highly selective Li^+/Mg^{2+} separation. *J. Memb. Sci.* **620**, 118862 (2021).
72. Xu, P. et al. "Bridge" graphene oxide modified positive charged nanofiltration thin membrane with high efficiency for Mg^{2+}/Li^+ separation. *Desalination* **488**, 114522 (2020).
73. Guo, C. et al. Ultra-thin double Janus nanofiltration membrane for separation of Li^+ and Mg^{2+}: "Drag" effect from carboxyl-containing negative interlayer. *Sep. Purif. Technol.* **230**, 115567 (2020).
74. Xu, P. et al. Positive charged PEI-TMC composite nanofiltration membrane for separation of Li^+ and Mg^{2+} from brine with high Mg^{2+}/Li^+ ratio. *Desalination* **449**, 57–68 (2019).
75. Zhang, H-Z., Xu, Z-L., Ding, H. & Tang, Y-J. Positively charged capillary nanofiltration membrane with high rejection for Mg^{2+} and Ca^{2+} and good separation for Mg^{2+} and Li^+. *Desalination* **420**, 158–166 (2017).
76. Li, W. et al. A positively charged composite nanofiltration membrane modified by EDTA for LiCl/$MgCl_2$ separation. *Sep. Purif. Technol.* **186**, 233–242 (2017).
77. Xu, P., Hong, J., Xu, Z., Xia, H. & Ni, Q-Q. MWCNTs-COOK-assisted high positively charged composite membrane: Accelerating Li^+ enrichment and Mg^{2+} removal. *Compos. Part B- Eng.* **212**, 108686 (2021).
78. Guo, C. et al. Amino-rich carbon quantum dots ultrathin nanofiltration membranes by double "one-step" methods: Breaking through trade-off among separation, permeation and stability. *Chem. Eng. J.* **404**, 127144 (2021).

79. Lu, D. et al. Constructing a selective blocked-nanolayer on nanofiltration membrane via surface-charge inversion for promoting Li^{+} permselectivity over Mg^{2+}. *J. Memb. Sci.* **635**, 119504 (2021).
80. Aghili, F., Ghoreyshi, A. A., Van der Bruggen, B. & Rahimpour, A. A highly permeable UiO-66-NH_2/polyethyleneimine thin-film nanocomposite membrane for recovery of valuable metal ions from brackish water. *Process Safe. Environ.* **151**, 244–256 (2021).
81. Xu, P. et al. Fabrication of highly positively charged nanofiltration membranes by novel interfacial polymerization: Accelerating Mg^{2+} removal and Li^{+} enrichment. *J. Memb. Sci.* **668**, 121251 (2023).
82. Wu, H. et al. Positively-charged PEI/TMC nanofiltration membrane prepared by adding a diamino-silane coupling agent for Li^{+}/Mg^{2+} separation. *J. Memb. Sci.* **672**, 121468 (2023).
83. Feng, Y., Peng, H. & Zhao, Q. Fabrication of high performance Mg^{2+}/Li^{+} nanofiltration membranes by surface grafting of quaternized bipyridine. *Sep. Purif. Technol.* **280**, 119848 (2022).
84. Li, H. et al. Nanofiltration membrane with crown ether as exclusive Li^{+} transport channels achieving efficient extraction of lithium from salt lake brine. *Chem. Eng. J.* **438**, 135658 (2022).
85. Wu, M-B. et al. Positively-charged nanofiltration membranes constructed via gas/liquid interfacial polymerization for Mg^{2+}/Li^{+} separation. *J. Memb. Sci.* **644**, 119942 (2022).
86. Peng, H. & Zhao, Q. A nano-heterogeneous membrane for efficient separation of lithium from high magnesium/lithium ratio brine. *Adv. Funct. Mater.* **31**, 2009430 (2021).
87. Yuan, B. et al. Aliphatic polyamide nanofilm with ordered nanostripe, synergistic pore size and charge density for the enhancement of cation sieving. *J. Memb. Sci.* **660**, 120839 (2022).
88. Ni, H., Wang, N., Yang, Y., Shen, M. & An, Q-F. Positively-charged nanofiltration

membrane constructed by polyethyleneimine/layered double hydroxide for Mg^{2+}/Li^+ separation. *Desalination* **548**, 116256 (2023).

89. Li, Y. et al. Polyamide nanofiltration membranes with rigid–flexible microstructures for high-efficiency Mg^{2+}/Li^+ separation. *Sep. Purif. Technol.* **306**, 122552 (2023).
90. Guo, C. et al. One-step construction of the positively/negatively charged ultrathin Janus nanofiltration membrane for the separation of Li^+ and Mg^{2+}. *ACS Appl. Mater. Inter.* **15**, 4814–4825 (2023).
91. Li, Y. et al. Fabrication of positively charged nanofiltration membrane with uniform charge distribution by reversed interfacial polymerization for Mg^{2+}/Li^+ separation. *J. Memb. Sci.* **659**, 120809 (2022).
92. He, R. et al. Polyelectrolyte-based nanofiltration membranes with exceptional performance in Mg^{2+}/Li^+ separation in a wide range of solution conditions. *J. Memb. Sci.* **663**, 121027 (2022).
93. He, R. et al. Unprecedented Mg^{2+}/Li^+ separation using layer-by-layer based nanofiltration hollow fiber membranes. *Desalination* **525**, 115492 (2022).
94. Shen, Q., Xu, S-J., Xu, Z-L., Zhang, H-Z. & Dong, Z-Q. Novel thin-film nanocomposite membrane with water-soluble polyhydroxylated fullerene for the separation of Mg^{2+}/Li^+ aqueous solution. *J. Appl. Polym. Sci.* **136**, 48029 (2019).
95. Zhao, J. et al. Mix-charged polyamide membranes via molecular hybridization for selective ionic nanofiltration. *J. Memb. Sci.* **644**, 120051 (2022).
96. Yuan, B. et al. Asymmetric polyamide nanofilm with coordinated charge and nanopore, tuned by azlactone-based monomer to facilitate ion separation. *Sep. Purif. Technol.* **304**, 122361 (2023).
97. Zhang, T. et al. Advanced Mg^{2+}/Li^+ separation nanofiltration membranes by introducing hydroxypropyltrimethyl ammonium chloride chitosan as a co-monomer. *Appl. Surf. Sci.* **616**, 156434 (2023).
98. Wu, H. et al. A novel nanofiltration membrane with [MimAP][Tf_2N] ionic liquid for utilization of lithium from brines with high Mg^{2+}/Li^+ ratio. *J. Memb. Sci.* **603**,

117997 (2020).

99. Li, X. et al. Preparation and characterization of positively charged polyamide composite nanofiltration hollow fiber membrane for lithium and magnesium separation. *Desalination* **369**, 26–36 (2015).

100. Bi, Q., Zhang, C., Liu, J., Liu, X. & Xu, S. Positively charged zwitterion-carbon nitride functionalized nanofiltration membranes with excellent separation performance of Mg^{2+}/Li^{+} and good antifouling properties. *Sep. Purif. Technol.* **257**, 117959 (2021).

101. Wang, L. et al. Novel positively charged metal-coordinated nanofiltration membrane for lithium recovery. *ACS Appl. Mater. Inter.* **13**, 16906–16915 (2021).

102. Ren, L., Chen, J., Han, J., Liang, J. & Wu, H. Anti-scaling covalent organic framework membranes with custom-tailored nanochannels for efficient lithium extraction. *Chem. Eng. J.* **462**, 142112 (2023).

103. Zhang, S. et al. Guanidyl-incorporated nanofiltration membranes toward superior Li^{+}/Mg^{2+} selectivity under weakly alkaline environment. *J. Memb. Sci.* **663**, 121063 (2022).

104. Xu, S. et al. Extraction of lithium from Chinese salt-lake brines by membranes: Design and practice. *J. Memb. Sci.* **635**, 119441 (2021).

105. Nightingale, E. R. Phenomenological theory of ion solvation. Effective radii of hydrated ions. *J. Phys. Chem.* **63**, 1381–1387 (1959).

106. Tansel, B. Significance of thermodynamic and physical characteristics on permeation of ions during membrane separation: Hydrated radius, hydration free energy and viscous effects. *Sep. Purif. Technol.* **86**, 119–126 (2012).

107. Yaroshchuk, A. & Bruening, M. L. An analytical solution of the solution-diffusion-electromigration equations reproduces trends in ion rejections during nanofiltration of mixed electrolytes. *J. Memb. Sci.* **523**, 361–372 (2017).
