# Supplementary material for: Self-assembled dendrimer polyamide nanofilms with enhanced effective pore area for ion separation
Source: Nat Commun. 2024 Jan 11;15:471. doi: 10.1038/s41467-023-44530-2 (PMC10784486; doi:10.1038/s41467-023-44530-2)
Supplement: Supplementary file 3 — Description of additional supplementary files [file 41467_2023_44530_MOESM3_ESM.pdf]

Description of additional supplementary files

Supplementary Software: Software and the related codes of a MATLAB application used for data fitting, and these data include ion permeability, water recovery, Li recovery and membrane area.
